# Differentiation of MIS 9 and MIS 11 in the continental record: vegetational, faunal, aminostratigraphic and sea-level evidence from coastal sites in Essex, UK

**DOI:** 10.1016/j.quascirev.2009.04.017

**Published:** 2009-11

**Authors:** Helen M. Roe, G. Russell Coope, Robert J.N. Devoy, Colin J.O. Harrison, Kirsty E.H. Penkman, Richard C. Preece, Danielle C. Schreve

**Affiliations:** a School of Geography, Archaeology and Palaeoecology, Queen's University of Belfast, Belfast BT7 1NN, UK; b Centre for Quaternary Research, Department of Geography, Royal Holloway, University of London, Egham, Surrey TW20 OEX, UK; c The Coastal Resources Centre, Department of Geography, University College Cork, Cork, Ireland; d Department of Zoology, Natural History Museum at Tring, Akeman Street, Tring, Hertfordshire HP23 6AP, UK; e BioArCh, Department of Chemistry, University of York, York YO10 5DD, UK; f Department of Zoology, University of Cambridge, Downing Street, Cambridge CB2 3EJ, UK

## Abstract

Multidisciplinary investigations of the vegetational, faunal and sea-level history inferred from the infills of buried channels on the coast of eastern Essex have a direct bearing on the differentiation of MIS 11 and MIS 9 in continental records. New data are presented from Cudmore Grove, an important site on Mersea Island that can be linked to the terrace sequence of the River Thames. The vegetational history has been reconstructed from a pollen sequence covering much of the interglacial represented. The temperate nature of the climate is apparent from a range of fossil groups, including plant remains, vertebrates (especially the rich herpetofauna), molluscs and beetles, which all have strong thermophilous components. The beetle data have been used to derive a Mutual Climatic Range reconstruction, suggesting that mean July temperatures were about 2 °C warmer than modern values for southeast England, whereas mean January temperatures may have been slightly colder. The sea-level history has been reconstructed from the molluscs, ostracods and especially the diatoms, which indicate that the marine transgression occurred considerably earlier in the interglacial cycle than at the neighbouring Hoxnian site at Clacton. There are a number of palynological similarities between the sequence at Cudmore Grove and Clacton, especially the presence of *Abies* and the occurrence of *Azolla filiculoides* megaspores. Moreover, both sites have yielded Palaeolithic archaeology, indeed the latter is the type site of the Clactonian (flake-and-core) industry. However, the sites can be differentiated on the basis of mammalian biostratigraphy, new aminostratigraphic data, as well as the differences in the sea-level history. The combined evidence suggests that the infill of the channel at Cudmore Grove accumulated during MIS 9, whereas the deposits at Clacton formed during MIS 11. The infill of a much later channel, yielding non-marine molluscs and vertebrates including *Hippopotamus*, appears to have formed during the Ipswichian (MIS 5e). This evidence is compared with other important sites of late Middle Pleistocene age in Britain and elsewhere on the continent and the importance of a multidisciplinary approach is stressed.

## 1 Introduction

The differentiation of MIS 9 from MIS 11 in the continental record is a topic of ongoing debate. In Britain there is a consensus in favour of correlation of the Anglian Stage with MIS 12 and of the ensuing Hoxnian Stage with MIS 11 (e.g. Bridgland, 1994; Bridgland et al., 1999; Rowe et al., 1999; Grün and Schwarcz, 2000; Schreve, 2001; Preece et al., 2007). However, in continental Europe, much uncertainty surrounds both the correlation of the Elsterian and the Holsteinian with other continental or deep-sea records. Like the consensus in Britain, many European workers favour correlation of the Holsteinian with at least part of MIS 11 (Sarntheim et al., 1986; Sibrava, 1986; Reille et al., 2000; de Beaulieu et al., 2001; Rousseau, 2003; Antoine and Limondin-Lozouet, 2004; Nitychhoruk et al., 2005, 2006). However, a recent study by Geyh and Müller (2005) has promoted correlation with MIS 9 based on some new TIMS ^230^Th/U dates from two peat layers from the Holsteinian reference site at Bosel in northern Germany and reappraisal of uranium series dates from two Hoxnian sites in England. They also reconsider the correlation of certain critical pollen sequences from maar lakes in France to support their case and they consequently dismiss the potential conflation of two “Holstein-like Interglacials” as “a problem confined to the Quaternary of the British Isles” (Geyh and Müller, 2006: p. 3072). Such divergent views hamper assessment of events within the late Middle Pleistocene.

The Thames Valley in southern England is one region where a range of deposits of late Middle Pleistocene age have been distinguished, largely because the critical interglacial sediments occur at different elevations in the terrace staircase (e.g. Bridgland, 1994). Pollen analysis, the technique most frequently used for biostratigraphy, often fails to discriminate between late Middle Pleistocene interglacials, especially in fluvial contexts, where the records are notoriously fragmentary and frequently undiagnostic (cf. Thomas, 2001). However, different stages can be distinguished, often fairly easily, using molluscan (Preece, 1995, 1999) and especially mammalian biostratigraphy (Schreve, 2001). By integrating terrace and faunal biostratigraphical data with aminostratigraphy, Bridgland (1994) identified four post-Anglian temperate stages in the Thames terrace record in the Lower Thames region downstream of central London, which he correlated with MIS 11, 9, 7 and 5e respectively. Moreover, new aminostratigraphic techniques, involving the analysis of multiple amino acids contained within the crystalline lattice of fossil shells (especially the calcitic opercula of the freshwater gastropod *Bithynia*), have provided additional independent support for such differentiation (Penkman, 2005; Penkman et al., 2007a).

**Fig. 1:**
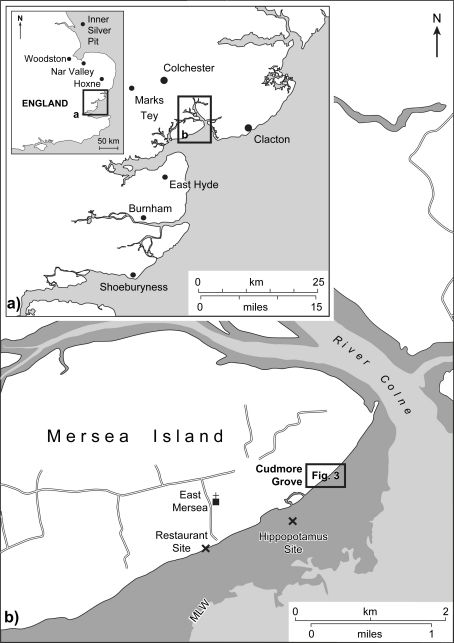
Location map of Cudmore Grove and the East Mersea Restaurant Site. The location of key interglacial sites in East Anglia referred to in the text is shown.

This paper reviews evidence from eastern Essex, on the western margin of the North Sea basin, that has a direct bearing on these issues. New data are presented from Cudmore Grove, a coastal site on Mersea Island, where a deeply-incised channel cut into London Clay (Eocene) has been infilled by interglacial estuarine silts and clays and now lies buried beneath fluvial gravels. The sediments, which cover a substantial part of the interglacial, can be linked into the terrace sequence of the Thames. Cudmore Grove is located just 10 km to the west of Clacton-on-Sea, the type site of the Lower Palaeolithic Clactonian industry (Oakley and Leakey, 1937), which has been correlated with the Hoxnian interglacial (Fig. 1; Pike and Godwin, 1953; Warren, 1955; Turner and Kerney, 1971; Miller et al., 1979; Bowen et al., 1989; Bridgland et al., 1999). The pollen records from these two sites bear a number of similarities and they were initially attributed to the same interglacial, namely the Hoxnian (cf. Bridgland, 1988, 1994; Roe, 1994). However, several lines of evidence suggest that this correlation is not secure. Despite their proximity, we show that the faunal records from the two sites differ significantly, as does their relative sea-level history. These differences indicate that the sites are not of the same age, a conclusion supported by the new aminostratigraphic data presented here that indicate that the sediments at Clacton formed during MIS 11 and those at Cudmore Grove during MIS 9. On the same foreshore, 2 km from the site at Cudmore Grove, another interglacial channel occurs now buried by modern beach shingle. This is the ‘East Mersea Restaurant Site’, which has yielded a limited fauna of non-marine molluscs and vertebrates, including *Hippopotamus* indicative of an Ipswichian age (correlated with MIS 5e) (Fig. 1). Thus channel sediments belonging to four different interglacials (including the Holocene), occur along this limited stretch of coastline. This provides an opportunity to examine faunal, sea level and other differences between the stages within a small geographical region and in particular to define the characteristics of MIS 9 in greater detail.

## 2 Regional setting

**Fig. 2:**
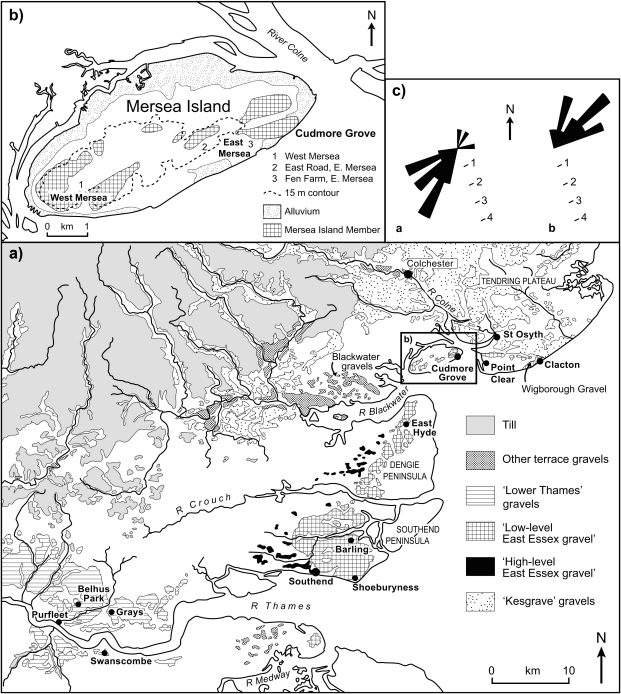
Distribution of sand and gravels associated with the Thames and its tributaries in eastern Essex and the eastern area of the Lower Thames region (after Bridgland, 1988, 1994). The distribution of Anglian till and the pre-Anglian gravels of the Thames (‘Kesgrave’ gravels) are indicated. For ease of interpretation, and to enable the various deposits to be related to the diversion of the Thames into the Southend and Dengie Peninsulas during the Anglian Stage, the terminology applied to the gravels follows Bridgland (1994). The pre-diversion deposits of the Essex Medway (‘High-level East Essex gravels’), the post-diversion Thames–Medway deposits (‘Low-level East Essex gravels’) and the Thames gravels of the Lower Thames area are all identified separately (cf. Bridgland, 1988, 1994). A more complete overview of the formal nomenclature applied to the gravels is given in the text and in Gibbard (1999). Inset (b) shows the distribution of the Mersea Island Member outcrops on Mersea Island (after Bridgland, 1983); inset (c) shows the palaeocurrent measurements from Units 6a (a) and 6b (b). The location of key sites mentioned in the text is indicated.

The coastal fringe of eastern Essex is dominated by a series of low-lying (<ca 30 m O.D.) Pleistocene terrace gravels that extend in a north-easterly direction between Southend and Clacton (Fig. 2). These deposits are largely of Thames origin and post-date the Anglian glaciation, when the Thames was diverted by ice from an earlier, more northerly course across central and northern Essex into the valley of an existing tributary, the Essex Medway (cf. Gruhn et al., 1974; Bridgland, 1983, 1994, Fig. 5.5; Gibbard, 1985, 1994). Older, pre-Anglian diversion Thames deposits, assigned to the ‘Kesgrave Member’ of the Lower Thames Formation (Gibbard, 1999) occur to the north of Mersea Island in the Tendring Plateau area, whilst pre-diversion gravels of the Essex Medway, the ‘High-level East Essex Gravels’ (Bridgland, 1994), survive as isolated remnants in the west of the coastal region (Fig. 2). The study area lies beyond the Anglian ice limits; the nearest glacial deposits occur a few kilometres to the west of Colchester (Fig. 2).

The terrace gravels of south-eastern Essex are locally underlain by remnants of a series of palaeo-channels cut into the London Clay (Lake et al., 1977; Bridgland, 1983, 1988, 1994; Roe, 1994, 1999). Of these, the channel-fill sequence at Cudmore Grove is the best preserved and most accessible. These features represent incised river channels of the diverted Essex Thames (or ‘Thames–Medway’) and its associated tributaries as the river migrated southwards across the region during successive warm stages of the late Middle Pleistocene (Bridgland et al., 1988, 1999; Bridgland, 1994; Roe, 1994, 2001). Palaeontological analyses have confirmed that the southeast Essex channel deposits accumulated under fully temperate conditions at times of high relative sea level (Warren, 1955; Bridgland, 1994; Roe, 1994, 1999, 2001; Bridgland et al., 1999). The terrace gravels and the channel-fill sediments are truncated locally by Holocene estuarine sediment, particularly around the east-trending estuaries of the Crouch, Blackwater and Colne (Fig. 2).

## 3 Field investigations

**Fig. 3:**
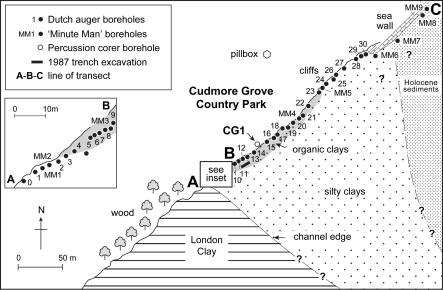
Location of boreholes at Cudmore Grove. The channel edge is indicated. The cross sections A–C and A–B refer to Figs. 4 and 5 respectively.

**Fig. 4:**
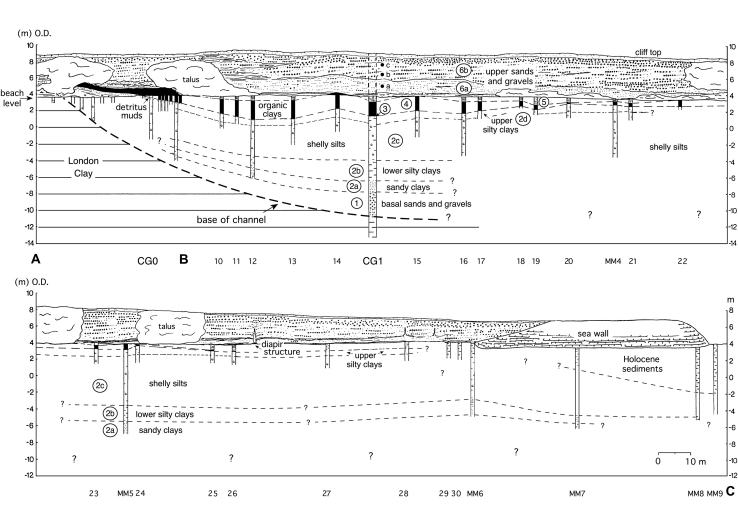
Section A–C showing the lithostratigraphy of the Cudmore Grove channel-fill sequence and overlying sands and gravels (Unit 6). The location of the boreholes is shown in Fig. 3. The position of Unit 3, only a few centimetres thick, at the base of the organic sequence (depicted in black) is indicated. The stratigraphical position of this unit is shown more clearly in Fig. 5. The lateral continuity of sub-units 2c and 2d near boreholes MM6–MM7 is unclear; here the Pleistocene beds are replaced by Holocene estuarine deposits (Fig. 3) which are compositionally similar to the upper part of the Pleistocene channel-fill sequence and locally indistinguishable. Samples for clast lithological analysis (Table 1) were collected from the cliff sections near borehole CG1 (sampling points a, b, c).

**Fig. 5:**
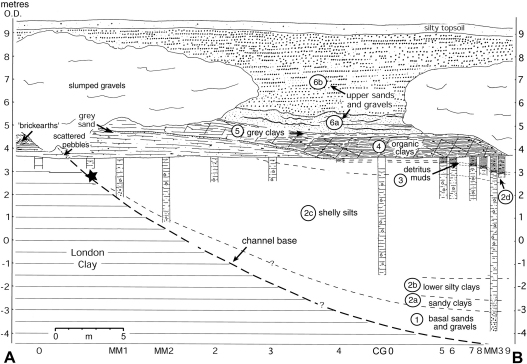
Channel margin, section A–B. The star symbol marks the position of the single struck flake found *in situ* in the Cudmore Grove Channel gravels (Unit 1).

The sedimentary sequence at Cudmore Grove was examined using a combination of surface mapping, section logging and coring techniques. The exposed parts of the sequence, which include sand and gravel cliffs and foreshore exposures, were mapped along a 0.3 km section extending between Cudmore Grove wood [TM 065 144] and the sea wall [TM 069 147] (Figs. 3–5). The subsurface stratigraphy was investigated using a series of shallow (ca 1–9 m) borings taken with a hand-auger and a ‘Minute Man’ portable drilling apparatus. A deep (22 m) borehole (CG1) was also excavated with a 10 cm diameter percussion corer to sample the complete Pleistocene sequence (Figs. 3 and 4). This was positioned on the cliff top at a point where the channel-fill sequence was estimated to be at maximum thickness. Bulk samples were obtained from a trench dug into the foreshore with a mechanical excavator (Fig. 3; see also Bridgland, 1994, Fig. 5.25).

## 4 Lithostratigraphy

With the exception of the sands and gravels exposed in the cliffs, which extend for several kilometres along the coast of Mersea Island (Fig. 3), the entire Pleistocene sequence at Cudmore Grove fills a steep-walled, channel-like depression cut into the London Clay, the ‘Cudmore Grove Channel’ (Roe, 1994) (Figs. 3–5). This feature is at least 0.28 km wide and 0.25 km long and is aligned in a northwest–southeast direction. The southern edge of the channel is well exposed at the base of the cliffs near Cudmore Grove wood and on the foreshore (Fig. 3). Its maximum observed depth was recorded in borehole CG1 at −10.40 m O.D. (Fig. 4). The depth further to the east is unknown, where the Pleistocene beds are truncated by Holocene sediment.

The Pleistocene sequence at Cudmore Grove can be divided into six units:

### Unit 1: Basal sands and gravels (Cudmore Grove Channel Gravel)

The floor of the channel is lined with sands and gravels that form the basal member of the Pleistocene sequence and directly overlie London Clay. These reach a maximum recorded thickness (3.10 m) in borehole CG1 (Fig. 4), then thin progressively south-westwards towards the channel margin, where they taper into a 1–2 cm pebble lag (Figs. 4 and 5), the ‘Cudmore Grove Channel lag’ (Bridgland et al., 1988). The persistence of Unit 1 to the northeast of borehole CG1 is unknown.

### Unit 2: Silty clays

The basal gravels grade upwards into a thick sequence of silts and clays, which occupy most of the channel and are exposed over wide areas of the foreshore. This unit can be divided into four sub-units:

#### a. Sandy clays

Massive, dark grey sandy clays occupy the basal 0.5–1 m. These reach maximum thickness (1.2 m) in borehole CG1, and thin towards the channel margin (Fig. 4). They can also be traced at the eastern end of the site below −5 m O.D. The continuity of the sub-unit beyond this point is unknown.

#### b. Lower silty clays

The sandy clays pass gradually upwards into brown-grey silty clays (Munsell: 10YR 4/1), commonly interbedded with thin, horizontal and gently inclined sand bands. Shell fragments occur occasionally. The lower silty clays reach maximum thickness (ca 3 m) between boreholes CG1 and MM5 and can be traced as far north as the sea wall (Fig. 4).

#### c. Shelly silts

The lower silty clays grade upwards into a thick sequence of massive, dark grey silts with scattered shell debris. These vary in thickness, from ca 5 m near borehole MM5 to <1 m in the southwest. The shelly silts can also be traced to the end of the sea wall in the northeast, where they extend from −3 m to 2 m O.D. (Fig. 4). The sub-unit is truncated further to the east by Holocene estuarine sediment.

#### d. Upper silty clays

Green-grey silty clays, (ca 0.5 m) with occasional horizontal sand beds form the uppermost member of this unit, which contains occasional scattered wood fragments and shell debris.

### Unit 3: Detritus muds

The silty clays are truncated at the southwestern part of the site by a thin bed of shelly detritus muds, composed of abundant shell debris in a matrix of dark brown (7.5YR 2/0) silty clay. This deposit, which when sampled in the early 1990s was only a few centimetres thick, is richly fossiliferous and has yielded a wealth of faunal remains (Holman et al., 1990; see below). The detritus muds were formerly more extensive and up to 30 cm thick (Bridgland et al., 1988) but have been eroded significantly by ongoing cliff retreat. The contact between the detritus muds and the silty clays of Unit 2 is sharp and is marked by a thin bed of coarse sand. The maximum thickness of the detritus muds (15 cm) was recorded near borehole MM3 (Fig. 5). From here the unit thins rapidly westwards, grading into 2–3 cm layer of grey sand and eventually disappearing near borehole MM5.

### Unit 4: Organic clays

**Fig. 6:**
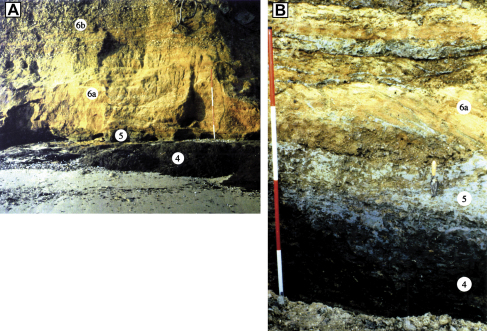
A) Photograph of an outcrop of the organic clays (Unit 4) at the base of the Cudmore Grove cliffs (facing north west), taken near borehole 14 (Fig. 4). The overlying sands and gravels (Unit 6) are sandier at the base (sub-unit 6a) and become clast-dominated in the upper part of the cliff exposure (sub-unit 6b). B) Photograph of the organic clays (Unit 4) and overlying grey clays (Unit 5) taken in the cliff sections near borehole 4 (Fig. 5).

Organic clays with abundant wood fragments overlie the detritus muds. The contact between the two units is abrupt and is marked by the sudden disappearance of shell debris. Like the detritus muds, the organic clays achieve maximum thickness (2.5 m) in the southwestern part of the site, where two facies can be recognised, a lower ca 20–50 cm bed of highly compressed wood interbedded with organic clay, and an upper 1–2 m bed of massive, organic clay with occasional wood fragments. The lower deposit is the more heterogeneous and is partly laminated. Three tree trunks have been exhumed from this horizon, one with its roots still intact (Bridgland, 1994, Fig. 5.26). Both beds show signs of compaction and local deformation. The organic clays thin steadily south-westwards, where they finally meet the rising surface of the London Clay. They also thin north-eastwards and eventually become truncated by the Upper sands and gravels (Unit 6) (Figs. 5 and 6A).

### Unit 5: Grey clays

Massive, grey clays, 5–30 cm thick, form the uppermost member of the channel-fill sequence (Fig. 6B). The contact between the two deposits is variable, sometimes it is marked by a transitional zone of brown-grey clay, but elsewhere it is abrupt. The unit extends laterally towards the eastern part of the site where it rests directly above Unit 2. It finally disappears near borehole 25 (Figs. 4 and 5).

Units 1–5 together complete the infill sequence of the Cudmore Grove Channel, which we formally define here as the ‘Cudmore Grove Member’ of the Lower Thames Formation (cf. Gibbard, 1999). Borehole CG1, which includes the thickest representation of most of the sedimentary units, is proposed as the stratotype [TM 0681 1456].

### Unit 6: Upper sands and gravels (Mersea Island Member, Lower Thames Formation)

The fine-grained beds are capped by 4–5 m of sands and gravels, which are exposed in the Cudmore Grove cliffs (Figs. 4, 5 and 6A). These were originally assigned to the ‘Mersea Island Gravel’ of the Low-level East Essex Gravel Formation (Bridgland, 1983, 1988, 1994), which comprised all the diverted Thames gravel aggradations in eastern Essex. This has since been re-termed the ‘Mersea Island Member’ of the Lower Thames Formation (Gibbard, 1999) with Cudmore Grove the designated type locality. Accelerated cliff erosion in recent years has provided excellent exposures of the sands and gravels that comprise two main facies: a lower 2–3 m suite of cross-bedded sands (Unit 6a); and an upper 3 m of mainly clast-supported gravels (Unit 6b) (Figs. 4 and 5). The lower unit, which forms a sharp contact with the underlying grey clays and organic clays, is best developed in the western part of the cliff exposure, where it comprises large-scale, gently dipping foresets of sand and subsidiary gravel. Individual foresets are 15–50 cm thick and dip at angles of 5–20°. Palaeocurrent measurements show that the prevailing flow direction was towards the north-north-east (Fig. 2c). In the lower part of this unit the sand-rich facies are locally interbedded with a series of thin (1–5 cm) grey clay horizons (Fig. 6B).

The sandy beds are truncated by the clast-rich facies of Unit 6b, which thicken progressively towards the sea wall. These too are cross-stratified, particularly in the lower 2 m, although the foresets are smaller than in Unit 6a. In contrast to the lower facies, palaeocurrent measurements show that flow was towards the southeast (Fig. 2c).

Unit 6b shows several signs of periglacial deformation, including a number of ice-wedge casts. At the southwestern end of the exposure the gravelly facies are also interbedded with large lobes of massive, heavily oxidized silty clay (‘brickearth’) (Bridgland, 1983; Bridgland et al., 1988; Fig. 5), thought to comprise soliflucted London Clay (Bridgland, 1994).

## 5 Clast lithological analysis

Clast lithological analysis has been an important tool in this region for distinguishing deposits of the Thames, which became confluent with the Essex Medway after its diversion into the region during the Anglian (forming the ‘Thames–Medway’), from older, pre-diversion Medway aggradations (Bridgland, 1983, 1988). The former contain clasts sourced from the wider catchment of the Thames, including the Weald, whereas the latter comprise lithologies originating from the London Basin (Bridgland, 1988). To ensure compatibility with previous work, samples were analysed using the technique described by Bridgland (1983: p. 25). Two samples were examined from the Cudmore Grove Channel Gravel (Unit 1, samples 1a and 1b) and three from Unit 6, the Upper sands and gravels (samples 6a–6c) (Fig. 4; Table 1). At least 400 stones were counted per sample. During counting, the pebbles were divided into three main categories: i) the ‘local’ group, which consists of rocks outcropping in the London Basin; ii) the ‘southern’ group comprising rocks occurring in the area to the south of the North Downs (including Lower Greensand chert); and iii) the ‘exotic’ group, comprising rocks from any other area and including exotic types reworked from within the London Basin (cf. Bridgland, 1983, 1988).

### Unit 1

The basal sands and gravels (Unit 1) recovered from borehole CG1 were found to comprise the same general lithologies (southern, exotic and local) as typical Thames–Medway gravels (Bridgland, 1988; Table 1). However, proportions of the three types differed significantly. First, the samples included much higher levels of angular (non-Tertiary flint), which gave Tertiary:non-Tertiary flint ratios of 5.36 and 6.22 (ratios from Thames–Medway gravels rarely exceed 1). These ratios are comparable to those recorded by Bridgland (1983) in the ‘Upper Holland Gravel’ near Clacton, a deposit that is thought to be enriched with distal outwash material (Bridgland, 1994: p. 287). The Unit 1 samples also yielded much higher frequencies of exotics (9.7% and 13.2% of the total) than local Thames–Medway gravels, which typically contain 0.5–2.5% (Bridgland, 1983). In this case, the most probable source is the exotic-rich Kesgrave Member deposits that outcrop widely to the north and west of Mersea Island (Fig. 2).

Overall, the range of lithologies in the Unit 1 gravels indicates a composition intermediate between Thames–Medway and outwash, with an additional element of Kesgrave material. The gravels may, therefore, have been aggraded by the Thames–Medway itself, downstream of a confluence with a river re-working fluvio-glacial sediments. Alternatively, they could have been deposited by a tributary that was fed by streams re-working older Thames–Medway, fluvio-glacial and Kesgrave deposits.

A single analysis from the Cudmore Grove Channel lag differed from the counts described above in showing a typical Thames–Medway composition (cf. Bridgland et al., 1988; Table 1). Unfortunately, no counts were obtained from this marginal deposit in the present study because pebble numbers were too low to obtain a valid sample size. Nevertheless, general observations confirmed that the deposit was indeed much richer in Tertiary flint than the gravels in borehole CG1. It therefore appears that the basal gravel is a composite deposit, the thinner, marginal part of which probably represents a lag derived from older Thames–Medway sediments.

### Unit 6

The upper sands and gravels (Unit 6) show some compositional similarities to the Thames–Medway gravels of the Southend and Dengie Peninsulas (cf. Bridgland, 1983; Gibbard et al., 1996). They differ, however, in their marginally higher proportions of exotics, which reach 2.4–4.6% (for comparison, frequencies in the Asheldham Member on the Dengie Peninsula rarely exceed 2.0%). The assemblages nevertheless compare closely with those of gravels found elsewhere on the island, which have also been assigned to the Mersea Island Member (Bridgland, 1983; Table 1). Bridgland (1983) attributed their slight enrichment of exotics to the input of additional quartzose material from a tributary, possibly the proto-Blackwater, which joined the Thames–Medway between the Dengie Peninsula and Mersea Island.

Whatever the origin of the exotics, the close similarities shown between the counts from Units 6a and 6b suggest that the same materials were being sourced throughout the aggradation, in spite of the inferred changes in flow direction (Table 1; Fig. 2c). This is somewhat surprising, since the upper gravel-rich facies appear to have been deposited by a river flowing from the northeast, a direction that is inconsistent with a Thames–Medway origin. It is therefore possible that these upper gravels were reworked by a smaller river (perhaps a proto-River Colne) from older Thames–Medway material to the north.

## 6 Regional distribution of the sediment sequence

The thickness of the sand and gravel overburden in the Cudmore Grove cliffs, (Fig. 4) renders it impossible to trace the Cudmore Grove Channel or its associated infill inland or immediately beyond the foreshore area. However, sand and gravel outcrops occur elsewhere on the island, which have been correlated with the gravel in the Cudmore Grove cliffs (Bridgland, 1983, 1988). Compositionally these gravels are similar to those at Cudmore Grove, although they differ considerably in height. The outcrops at West Mersea, for example, have basal elevations of ca 10–13 m O.D. (Bridgland, 1983), some 10 m higher than the gravels at Cudmore Grove (Figs. 2 and 4). These outcrops rest directly on London Clay. The outcrops near East Mersea are less well documented, although Bridgland (1983) established that the small outcrop to the west of the village (Fig. 2b) has a base of ca 10 m O.D. Sand and gravel outcrops at Point Clear, 4 km to the east of Cudmore Grove on the Tendring Plateau (Fig. 2), have also been assigned to the Mersea Island Member (cf. Bridgland, 1994).

Bridgland (1994) showed that the various outcrops of the Mersea Island Member correspond well, both in thickness and height, with the sands and gravels of the Asheldham Member (cf. Gibbard, 1999) on the Dengie Peninsula, which he considered its upstream equivalent. The Wigborough Gravel, ca 8 km to the east in the Clacton area (Fig. 2), was thought to represent the downstream equivalent on the Tendring Plateau (see Bridgland, 1994: p. 294 for a full account of the correlations). Like the gravels in the Cudmore Grove cliffs these also include slightly enriched levels of exotics (Bridgland, 1994: p. 322).

In spite of these long profile projections, it remains difficult to establish whether all the outcrops of the Mersea Island Member represent part of a single aggradation or whether they are composite in age. It is possible, for example, that some of the more elevated outcrops are older than those in the Cudmore Grove cliffs and may even pre-date the Cudmore Grove Channel and its infill. Relative age determinations based on long profile projections are also hampered, in this instance, by the fact that at least part of the Cudmore Grove cliff gravels sequence appears to have been deposited by a tributary river or rivers (the proto-Blackwater and/or proto-Colne) and not by the Thames–Medway (Roe, 1994). The stratigraphical and palaeogeographical implications of these findings are considered further below.

## 7 Pollen and plant macrofossils

### 7.1 Sampling and preparation

**Fig. 7:**
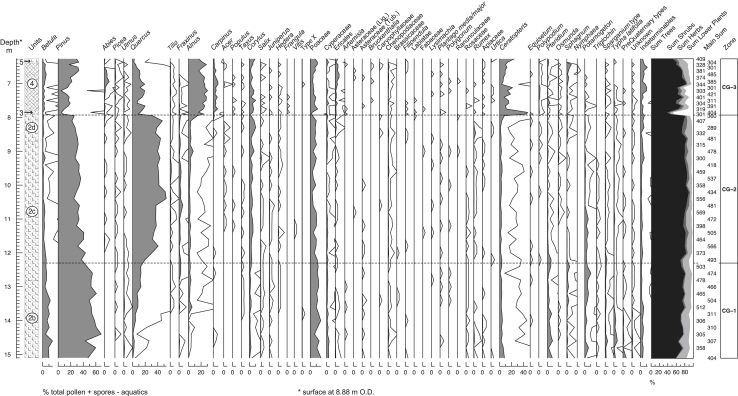
Pollen percentage diagram from core CG1. 10× exaggeration lines are indicated.

Pollen analyses were undertaken on the sediments of borehole CG1 and were prepared using the method of Berglund and Ralska-Jasiewiczowa (1986). Pollen was recovered from the sediments between 6.27 and 14.80 m in this borehole, spanning Units 2–4 (Fig. 7). The sandy facies at the base of Unit 2 (14.80–16 m) yielded only degraded *Pinus* pollen at sparse concentrations, precluding detailed analyses. Plant macrofossil remains retained at the sieving stage of pollen preparation are given in Table 2.

### 7.2 Pollen assemblage biozones

The pollen percentage diagram (Fig. 7) is divided into three pollen assemblage biozones, labelled with the prefix ‘CG’.

#### CG-1 (14.80–12.30 m)

This basal zone occurs in the sediments of sub-units 2b–2c. The spectra are dominated *Pinus* pollen, which rises to 69% at 14.40 m. *Betula* pollen also peaks in the lower section, then falls gradually near the upper zone boundary. *Quercus* pollen increases steadily through the zone. Of the other tree taxa, *Alnus* pollen frequencies are most significant, rising in the middle of the zone. Shrub taxa are poorly represented, with *Corylus* the most persistent. Poaceae and Cyperaceae pollen dominate the herbaceous taxa. Lower plant and aquatic pollen types are well represented, attaining peak frequencies of ca 15%.

With the exception of several *Typha* fruits, plant macrofossils were rare. However, several macrofossils were recovered from the underlying sandy sediments, which contained little or no pollen. These included two fruits of *Elatine hydropiper* (at 15.87 and 16.00 m) and an achene of *Ranunculus*, subgenus Batrachian (at 15.87 m) (Table 2).

#### CG-2 (12.30–7.93 m)

Sub-units 2c and 2d continue to the top of this zone. The base of the zone is marked by a sudden rise in the frequency of *Quercus* and a small increase in *Corylus* pollen. These trends continue to the middle of the zone, where *Quercus* achieves peak frequencies (53%). *Pinus* and *Betula* pollen frequencies decline simultaneously. The frequencies of most taxa stabilise in the upper half of the zone, with *Quercus* and *Pinus* dominant. *Alnus* and *Corylus* pollen undergo slight frequency increases. A single *Typha* fruit (at 12.20 m) and a bryophyte fragment (at −10.0 m) were the only macrofossils recorded.

#### CG-3 (7.93–6.27 m)

The base of this zone coincides with the appearance of the organic clays (Unit 4). The basal few centimetres contain sand and shell debris, representing the thin remnant in this core of Unit 3 (Fig. 7). The pollen spectra undergo many changes, which begin with a sharp peak in *Ceratopteris* spores and a steep rise in *Alnus* pollen. These changes coincide with a sharp fall in *Pinus*, *Quercus*, *Corylus* and Poaceae pollen. *Alnus* frequencies continue to rise in the central part of the zone, whereas *Ceratopteris* spores decline. *Pinus*, *Quercus* and *Corylus* pollen frequencies recover slightly, together with Poaceae and *Betula*. *Carpinus*, *Abies* and Ericales pollen frequencies also increase in the middle part of the zone. With the exception of *Pinus* and *Alnus*, frequencies of all the major arboreal taxa decrease at the top of the zone to <3%. These changes coincide with another sedimentary change, as the organic clays are replaced at 6.4 m by the grey clays (Unit 5). *Ceratopteris* spore frequencies show another sharp increase at this point. Only two samples from this unit yielded pollen.

The samples from this zone yielded abundant macrofossils, including several megaspores of *Azolla filiculoides* and three achenes of *Eupatorium cannabinum* (Table 2). A tree trunk (over 2 m long and probably *in situ*) and several other large pieces of *Alnus* wood were recovered from the basal part of the organic sequence elsewhere at the site.

### 7.3 Vegetation and local environment

The absence of pollen in the basal, sand-rich sediments of sub-unit 2a suggests that conditions were initially unfavourable for pollen sedimentation or preservation. However, the fact that these deposits yielded waterside herb macrofossils shows that marshland communities were already established. The occurrence of fruits of *E. hydropiper* is noteworthy in implying that interglacial conditions had already become established when Unit 2a accumulated. This species flourishes today in eutrophic, sandy-bottomed, shallow-water habitats with a thin cover of silt and organic detritus (Brinkkemper et al., 2008).

#### CG-1

The sediments and other microfossils (see below) associated with this zone suggest that a fairly substantial, tidally-influenced river channel now existed. The pollen source area is likely to have been large, including inputs from fluvial and limited marine sources. Boreal-type woodland prevailed in the regional catchment at the start of this phase; *Betula* and *Pinus* were both widespread, although the latter is likely to be overrepresented due to the buoyancy properties and high pollen productivity of *Pinus* grains (cf. Fægri and Iversen, 1989; Roe and van de Plassche, 2005). Oak, elm, ash and hazel persisted on a limited scale.

The composition of the forest changed subsequently as oak, and then ash, began to expand, out-competing birch in some areas. With this expansion came the colonisation of *Hedera* into the forest understorey. The unidentified palynomorph Type X first appears in this zone. This taxon has been reported from several Hoxnian sites (Thomas, 2001) and is believed to have been produced by a forest shrub, possibly a member of the Oleaceae (Turner, 1970). *Alnus* too became established, probably near the river margins.

Herb communities were fairly diverse throughout this period and point to the existence of lightly shaded forest-floor habitats or more open areas of grassland. *Pteridium* and *Sphagnum* confirm the development of damp, slightly acidic soils in parts of the region. Waterside and marsh plants also formed an important part of the flora during this time, probably flourishing near the river. These included sedges, ferns and several marshland herbs (*Typha latifolia*, *Filipendula*, *Sparganium* and *Lysimachia*). The *Potamogeton* pollen record shows that relatively calm, freshwater habitats prevailed locally.

#### CG-2

The sediments and other microfossil datasets suggest that marine influence became more pronounced as saltwater inflow gradually increased (see Sections 8–10 below). An enlargement of the pollen catchment can thus be inferred, as pollen was now carried in from estuarine and marine sources. The sudden increase in *Quercus* pollen at the start of the zone may partly reflect this taphonomic change, as *Quercus* pollen was introduced from coastal waters. In spite of this overrepresentation, oak was clearly becoming well established in the region, initiating a decline in both birch and pine. Pine probably persisted in some areas but on a reduced scale. *Fraxinus* and *Tilia* also expanded in the middle of the zone, reflecting the development of well-drained, base-rich soils within the catchment. This was accompanied by the gradual expansion of *Corylus* and the first arrival of *Vitis*, a shrub of damp, woodland habitats (Turner, 1968, 1970).

Some open areas persisted during this zone, supporting such genera as *Polygonum* and Caryophyllaceae. Some of the Chenopodiaceae and *Plantago* pollen may have been carried in from local saltmarshes. Waterside and aquatic plants declined, probably reflecting the continued salinity rise.

#### CG-3

The onset of zone CG-3 coincided with a marked change in depositional environment, as the estuarine channel regime of the previous zone was replaced by a much quieter phase of organic clay deposition, associated with a lagoonal backwater. This was associated with the deposition of Unit 4. A thin bed of sand (Unit 3, the detritus muds) accumulated in the intervening period, probably as a lag deposit following a short phase of denudation. This sequence of events appears to have initiated a dramatic change in pollen source area, as the input of regionally derived estuarine pollen (mostly *Quercus* and *Pinus*) ceased and was replaced by a strong local pollen signal. The influx of *Ceratopteris* spores at the start of zone shows that fern communities colonised the site, probably spreading over the damp, exposed surface of the infilled channel. Some of these resistant spores may also have been reworked.

Alder now dominated the local woodland and even became rooted at the site, showing that local soils were fairly wet. Drier areas, probably fringing the alder carr, still supported mixed oak woodland during the early part of the zone, with the same suite of taxa that flourished previously. This soon changed, however, as firstly *Carpinus* and then boreal trees (*Abies*, *Pinus* and *Betula*) began to expand. *Picea* was the only conifer not to invade, possibly because of the high clay content of the local soils (cf. Turner, 1970). The ground vegetation also changed, as Ericaceous plants became more widespread, probably in response to soil acidification.

Wetter areas continued to support *Alnus* in the latter half of the zone, although there was no longer any input of *Alnus* wood to the sediment, possibly because the water was now too deep for the tree to grow. Conditions were now more favourable to waterside, aquatic and semi-aquatic herb communities, including *E. cannabinum*, *A. filiculoides*, *Chara* and *Alisma*. All these plants prefer fairly quiet, eutrophic, freshwater conditions, although with the exception of *Azolla*, all could tolerate low levels of salt. *Sphagnum* too became more widespread, reflecting increasing dampness.

Conditions changed again near the end of the zone as the input of organic material ceased and inorganic clays and sands (Unit 5) began to accumulate. *Pinus*, *Alnus* and *Ceratopteris* were still present during this phase, although *Abies* appears to have declined in the immediate region. The thermophilous trees also diminished, possibly in response to a progressive shift towards boreal woodland. Because only the more resistant pollen grains are represented in the two uppermost samples it is possible that some of the pollen has been reworked, probably from the underlying organic beds.

### 7.4 Pollen biostratigraphy

The pollen sequence from Cudmore Grove shows two clear phases of vegetational development. The first, in biozones CG-1 and CG-2, records the regional establishment and expansion of mixed oak woodland, during the mesocratic phase of an interglacial, when closed forests developed under temperate conditions and soils became more fertile (cf. Birks and Birks, 2004). Biozone CG-3 in contrast, records the local development of alder carr woodland with a peripheral expansion of *Carpinus* and *Abies*. In northwest Europe the expansion of these two trees occurred in the oligocratic phase, when soils deteriorated and forest cover declined. In Britain, this broad pattern of vegetational succession has commonly been divided into four zones: the ‘pre-temperate’, ‘early-temperate’, ‘late-temperate’ and ‘post-temperate sub-stages’ (Turner and West, 1968). More complete palynological records from central and southern Europe (e.g. Roucoux et al., 2008) indicate that some interglacials have more complex histories of forest change, but we retain these terms here in order to facilitate comparisons of the pollen records with those of other Middle Pleistocene sites and as a framework for the discussion of the relative sea-level history.

The representation of pollen spectra of both early and late-temperate sub-stage character at Cudmore Grove is highly unusual within the context of the Thames region, where interglacial pollen records are typically fragmentary. A hiatus probably occurs between biozones CG-2 and CG-3, but there are strong grounds for believing that both of these phases date from the same interglacial. There is, for example, no evidence for a period of weathering or a major unconformity between the two biozones, or any evidence for an intermediate deterioration of climate. Alder also appeared to have been expanding locally at the end of zone CG-2 and continued to expand in CG-3, suggesting that the two biozones date from the same temperate episode. The profile from Cudmore Grove thus represents one of the most complete, non-lacustrine interglacial pollen records from Britain and is arguably the most important from the Thames system. However, given the changes in pollen source area and the estuarine/lagoonal context of the sediments, correlations with other sites, particularly records from lakes, must be made with caution.

Despite this difficulty, a number of palaeobotanical elements strongly support correlation with a ‘Hoxnian-type’ interglacial (cf. Scourse et al., 1999; Thomas, 2001). This evidence is strongest in the upper part of the sequence (biozone CG-3), where the presence of Type X and *Abies* pollen and megaspores of *A. filiculoides* together preclude correlation with any other interglacial sequences of the British Late-, Early and Middle Pleistocene. All three floristic elements were absent, for example, in the Ipswichian (=Eemian/MIS 5e) (cf. West, 1980). These elements are also absent at sites that have been correlated on the basis of their terrace stratigraphy, faunal assemblages or aminostratigraphy with MIS 7, for example, Aveley and Ilford in the Lower Thames region (Bridgland, 1994), although the pollen records from these sites are far less complete (West et al., 1964; West, 1969). Sparse *Abies* pollen (<0.5–1% of the total land pollen) has been reported from fluvial deposits which have been correlated with MIS 7 at Stoke Goldington (Green et al., 1996) and Marsworth (Murton et al., 2001) in Buckinghamshire, although these can be attributed to long-distance inputs (cf. Green et al., 1996). The spectra from both of these sites differ from those of Cudmore Grove in several other respects, particularly in their absence of Type X and their inclusion of greater frequencies of Poaceae pollen, which point to an incomplete regional tree cover in southern Britain at least during part of this interglacial.

More detailed correlations between the Cudmore Grove pollen assemblages and those from other ‘Hoxnian-type’ sites in Britain and continental Europe are hampered by several problems, not least the taphonomic issues outlined above, and the fact that more than one ‘Hoxnian-type’ temperate episode may have occurred with similar floristic characteristics (cf. Scourse et al., 1999; Thomas, 2001; Scourse, 2006). It is nevertheless appropriate to make brief comparisons with the records from three key sites in the immediate vicinity (<20 km) that have also yielded pollen assemblages of ‘Hoxnian’ character: i) the Hoxnian parastratotype succession at Marks Tey, Essex, a lacustrine sequence infilling a depression cut in Anglian till (Turner, 1970); and ii) the pollen spectra from the neighbouring channel-fill sequences at Clacton (Pike and Godwin, 1953; Bridgland et al., 1999) and Tillingham (East Hyde) (Roe, 2001) (Fig. 1). The last two channel-fills were deposited by the Thames in the interglacial that immediately followed the diversion of the river into eastern Essex during the late Anglian, i.e. the Hoxnian (cf. Bridgland, 1994; Bridgland et al., 1999; Roe, 2001). The sediments at all three sites have been correlated with MIS 11 (Bridgland et al., 1999; Rowe et al., 1999).

The Cudmore Grove pollen spectra share several attributes with those from Marks Tey, only 16 km to the northwest (Turner, 1970: p. 396; Fig. 7). Biozones CG-1 and CG-2, for example, compare closely with the spectra assigned to Ho IIa at this site, which are characterized by a sharp rise in *Quercus* pollen, a gradual expansion of *Alnus* and *Corylus* and persistent presence of *Fraxinus* pollen (Turner, 1970), although this sub-stage is not especially diagnostic (Thomas, 2001). The Cudmore Grove pollen profile differs from that of Marks Tey in the high frequencies of *Pinus*, although this might be explained by taphonomic differences between the sites.

Biozone CG-3 at Cudmore Grove is also broadly similar to the late-temperate sequence (Ho III) at Marks Tey, particularly in its record of *Abies*, *Carpinus* and Type X pollen. However, all three taxa achieve much greater abundances at Marks Tey than at Cudmore Grove, with *Abies* in particular peaking at frequencies of 45%, as opposed to ca 6% at Cudmore Grove (Fig. 7). This attribute alone does not provide sufficient basis for assuming that the sites are of different ages; the comparatively modest *Abies* profile at Cudmore Grove could be an artifact of the local dominance of *Alnus*, which may have obscured some of the regional vegetational changes upon which zonation of this sub-stage has been based (cf. West, 1956; Turner, 1970). *Abies* prefers drier soils and is thus unlikely to have flourished around Cudmore Grove at any time during the high *Alnus* phase. Moreover, even if *Abies* was widespread in the hinterland, it is doubtful whether much of its pollen would have reached the site, given its low rates of pollen productivity and restricted patterns of dispersal (Reille, 1990). *Taxus* pollen is also far more strongly represented at Marks Tey than at Cudmore Grove, achieving frequencies of ca 10% (Turner, 1970), although varying frequencies of yew pollen could reflect local differences in soil conditions (cf. Kelly, 1964). Unlike Marks Tey, there is also no record of *Pterocarya* in the late-temperate sub-stage at Cudmore Grove (cf. Turner, 1970).

The pollen profiles from Clacton and East Hyde (Tillingham) also include far greater abundances of *Abies* and *Carpinus* pollen than Cudmore Grove, although in other respects the assemblages are broadly similar, and all three sites have yielded Type X pollen and remains of *Azolla*. At Clacton, *Abies* peaks at ca 65% (Pike and Godwin, 1953), whilst at East Hyde a peak of 40% is recorded (Roe, 2001). This difference might again reflect taphonomic variations between the sites; during the late-temperate sub-stage of the interglacial represented at Cudmore Grove a sheltered lagoon appears to have existed, whereas the channels at East Hyde and Clacton were more open to estuarine influence during the corresponding interval (Pike and Godwin, 1953; Bridgland et al., 1999; Roe, 2001). This might have provided suitable conditions for the selective accumulation of bisaccate grains.

The palaeobotanical data from Cudmore Grove thus display broad similarities with the neighbouring records from Marks Tey, East Hyde and Clacton, but there are a number of subtle differences, particularly the differing proportions of *Abies* recorded in the late-temperate sub-stage. These may either reflect taphonomic differences between the sites, or may imply that the Cudmore Grove profile belongs to a younger interglacial also characterized by ‘Hoxnian-type’ vegetation, and possibly co-eval with MIS 9 (cf. Scourse et al., 1999; Thomas, 2001). Wider comparisons between the Cudmore Grove pollen record and sites in southern Britain and elsewhere are considered further below in the light of the other bio- and aminostratigraphic data.

## 8 Diatoms

### 8.1 Sampling and preparation

**Fig. 8:**
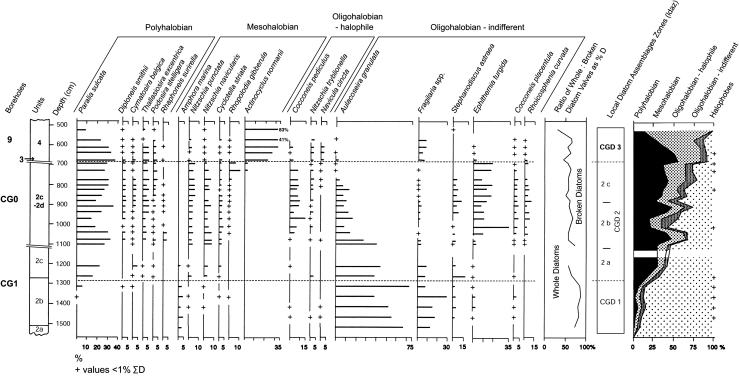
Composite diatom percentage diagram (selected taxa) based on samples from cores CG0, CG1 and a core collected from the organic clays and detritus muds (Units 3 and 4) near the point of borehole 9 (Fig. 5). The break recorded between 1100 cm and 1200 cm is an artifact of the change in cores at this point. Salinity groupings follow Hustedt (1957).

Samples for diatom analysis were taken from boreholes CG0 and CG1. Borehole CG0 (Fig. 5), collected in 1986, recovered ca 5.5 m of sediment spanning sub-units 2b–2d, below 0.5 m of modern beach sand. Samples from the eroded Units 3–4 at CG0 were taken from a separate borehole ca 8 m away, near the position of borehole 9 (Fig. 5). Borehole CG1, ca 50 m east of CG0 in the deeper part of the channel, which was collected in 1990, provided a thicker and more complete sedimentary sequence (Units 2–4) through the interglacial (Fig. 4). Diatom samples from this core came from 6.7 m to below 16 m, providing overlap and continuation of the record from CG0. The diatom assemblages from the cores have been integrated. Preparation of the sediments for diatoms was based upon standard techniques (Battarbee, 1986). Identification of diatoms, together with interpretations of their taphonomy and palaeoecology were based on established floras and other texts (e.g., van der Werff and Huls, 1957–1974; Admiraal, 1984). Results are presented as a percentage frequency diagram showing key species within salinity groupings (Fig. 8). The diatoms were generally well preserved and included >220 taxa.

### 8.2 Diatom assemblages

Three main local diatom assemblage zones (ldaz) are recognised (CGD1–CGD3; Fig. 8). In ldaz CGD2 the assemblage is complex and the zone has been divided into three subzones ldaz CGD2a–2c.

#### ldaz CGD1 (15.10–12.70 m)

This zone, which spans the lower silty clays (sub-unit 2b), is characterized by a fining upward sedimentary sequence. Diatoms are absent below ca 15 m. Freshwater (oligohalobous–indifferent) taxa, dominated by *Aulacoseira granulata* (Ehr.) Simonsen reach >50% of the total assemblage, with *Fragilaria pinnata* Ehr., *Fragilaria brevistriata* Grun. and *Fragilaria lapponica* Grun. together at >15%. Brackish-water (mesohalobous and oligohalobous–halophilous) species are present throughout the zone at low values (<10%). Marine diatoms (polyhalobous) occur erratically, with the littoral species *Paralia sulcata* (Ehr.) Cleve making an early appearance.

#### ldaz CGD2a (12.70–11.00 m)

This subzone begins close to the contact of sub-units 2b and 2c in borehole CG1 and marks the major expansion of polyhalobous taxa to >28%, with *P. sulcata* rising to >20%. Other marine taxa present at lower frequencies include *Diploneis smithii* (Bréb.) Cleve, *Cymatosira belgica* Grun. and *Thalassiosira excentrica* (Ehr.) Cleve. The expansion of marine diatoms is accompanied by a increase in brackish species, dominated by *Nitzschia punctata* (W. Smith) Grun., *Nitzschia navicularis* (Bréb.) Grun. and *Cyclotella striata* (Kütz.) Grun. Freshwater taxa remain dominant in ldaz CGD2a at >50%.

#### ldaz CGD2b (11.00–8.60 m)

Significant fluctuations occur between freshwater and marine diatoms in this subzone. Marine and brackish-water taxa continue to increase in abundance and species diversity, together reaching >60%. Freshwater, halophilous species also increase in importance, especially *Cocconeis pediculus* Ehr. and *Nitzschia tryblionella* Hantzsch. Freshwater taxa (oligohalobous–indifferent) generally represent <35% of the total assemblage. *Fragilaria* spp. fall through ldaz CGD2a to low values in ldaz CGD2b (<3%). *A. granulata* declines to 10–15% and is joined at similar values by *Epithemia turgida* (Ehr.) Kütz, accompanied by lower frequencies of *Stephanodiscus astraea* (Ehr.) Grun.

#### ldaz CGD2c (8.60–6.90 m)

The ‘peak and trough’ behaviour of the major marine and freshwater groups ceases. Marine and brackish-water taxa expand to >70%, with the mesohalobous and halophilous groups together reaching ca 35%. Freshwater diatoms continue to decline resulting in a community now characterized by *E. turgida*, and lower values of *Cocconeis placentula* Ehr. and *Rhoicosphenia curvata* (Kütz) Grun.

#### ldaz CGD3 (6.90–5.30 m)

The base of this zone marks the boundary between the silty clays (Unit 2d) and the organic beds (Units 3 and 4). Mesohalobous and halophilous diatoms expand in frequency to >75% of the total assemblage, dominated by the rapid rise of *Actinocyclus normanii* (Greg.) Hust., which reaches >80%. At the base of the zone, marine taxa, dominated by *P. sulcata* and *Th. excentrica*, rise to maximum values (ca 55%), before falling to ca 12% at the top of the zone. Freshwater diatoms show low, erratic frequencies and are dominated by the return of *Fragilaria* spp. to values of ca 8%.

### 8.3 Local environment inferred from the diatoms

Changes in the diatom assemblages show the probable long-term landward progression of the tidal head and accompanying salt wedge of a palaeo-estuary, with the development of full brackish-water conditions by ldaz CGD2c. Shorter term changes in the diatoms may represent i) major fluctuations in freshwater discharge to the estuary; ii) rises of relative sea level; and iii) sedimentary and/or estuarine–coastal geometry changes. Together these eventually led to the replacement of an estuarine tidal channel and mudflat environment by more isolated conditions, possibly an estuarine embayment or lagoon. Detailed diatom records of this kind are rare from British interglacial sites, although Mitlehner (1992) provides a skeletal record from the Nar Valley Clay in Norfolk (see Section 16.3 below).

During the early phase of channel infilling, associated with ldaz CGD1, the dominance of *Aulacoseira* spp. and *Fragilaria* spp. and the high values of whole diatoms (ca 80%), suggest that eutrophic, freshwater conditions prevailed locally, indicative of a riverine environment with adjacent alkaline freshwater areas. The presence of brackish and marine taxa reflects the penetration of saltwater into the river. *F. pinnata* and *F. lapponica* (each rising to >12%), have a northerly distribution (de Wolf and Cleveringa, 1994) and the diatom community is consistent with stressed conditions during the early part of an interglacial. *Fragilaria* spp. are pioneer diatoms that thrive under conditions of environmental stress, particularly where increasing salinity is involved (Stabell, 1985; Denys, 1990), as is *A. granulata* (Stachura and Witkowski, 1997). The assemblage as a whole is representative of the upper reaches of a river estuary close to the tidal limit, with salinities of ca 0.5–5‰.

The rapid rise in marine species in ldaz CGD2a, dominated by *P. sulcata* and other littoral and lower estuary taxa, shows the influence of tidal action. The rise in broken diatoms over the ldaz CGD1/2a boundary coupled with an increase in species diversity, together suggest the transport and inwashing of diatoms from a range of salinity regimes. The fining in sediment within Unit 2 may be associated with increasing salinity, resulting in flocculation. The continuing dominance of freshwater diatoms indicates that the site remained close to the tidal head in the river.

Above ca 11 m (CGD2b) the diatom flora reflects the effects of the developing estuary, shown by the expansion of the number and frequencies of brackish-water taxa. In the freshwater diatom community, the dominance of *A. granulata* and other planktonic taxa is replaced by benthic, epipelic taxa, especially, *E. turgida*. This indicates an increase in the extent of shallower, estuarine margin habitats. The fluctuations in marine to freshwater diatoms in ldaz CGD2b also indicate a period of variability in the environmental controls operating in the estuary. The occurrence of such fluctuating freshwater: marine diatom ratios in some estuarine records have been attributed to the effects of storms, river floods and marine surges, combined with the impact of longer term relative sea-level rises (Devoy et al., 1994). The record here shows that the ‘cycles’ in the diatom ratios each represent ca 0.5 m of sediment accumulation with no apparent hiatuses. This sediment thickness, together with its fine texture, suggest that these ‘cycles’ are unlikely to be the result of storms, which might be expected to have produced coarser layers, although it is possible that the channel margin location (for CG0) may have influenced the diatom pattern concentrating diatoms from many habitats and sources. Longer term controls (10^1^–10^2^ years) are more likely to have caused the observed changes. These include climate variability, leading to changes in freshwater river discharge, and alterations in the tidal prism as the estuary shape changed with rising relative sea level and sediment deposition.

The disappearance of these ‘cycles’ from the diatom record above ca 850 cm coincides with the steady expansion in the importance of brackish-water and marine diatoms. The maintenance of *N. punctata*, *N. navicularis*, *C. pediculus* and other epipelic – epiphytic mesohalobous and halophilous diatoms, indicates the occurrence of mudflat and intertidal conditions. The rising values of *C. belgica*, accompanied by many other marine planktonic and tychoplanktonic diatoms at the base of ldaz CGD2c, point to wave and tidal scouring (Vos and de Wolf, 1993) and the proximity of open coastal sandy areas. The site at this time was probably in a lower estuarine setting. The observed changes probably reflect the landward shift in estuary zones with long-term sea-level rise, causing increased exposure to marine influence.

The base of ldaz CDG3 is marked by an increase in broken diatoms and a peak in marine diatoms. The sediments also become more sandy, suggesting an increase in available energy. These samples came from Unit 3, which has yielded rich mammal and molluscan assemblages (see below). Given that these sediments appear to represent a lag or a high-energy flood deposit, erosion and/or re-working of diatoms from proximal marine sources seems likely.

The subsequent collapse of both the marine and freshwater diatom frequencies through ldaz CGD3, suggests a distinct change in conditions, as supported by the development of the organic clays (Unit 4). The rapid rise in *A. normanii* coupled with *Fragilaria* spp., indicates increased water eutrophication and environmental stress (cf. Stabell, 1985; Denys, 1990). The diatom flora represents the maintenance of fresh-brackish to brackish-water, indicative of isolated or semi-enclosed areas of standing water. This interpretation is consistent with the isolation of the site from the open estuary. The sediment infilling and shallowing of the estuary through the interglacial may now have led to the development of channel cut-offs, sediment barriers and estuarine lagoons.

## 9 Ostracoda

### 9.1 Sampling and preparation

**Fig. 9:**
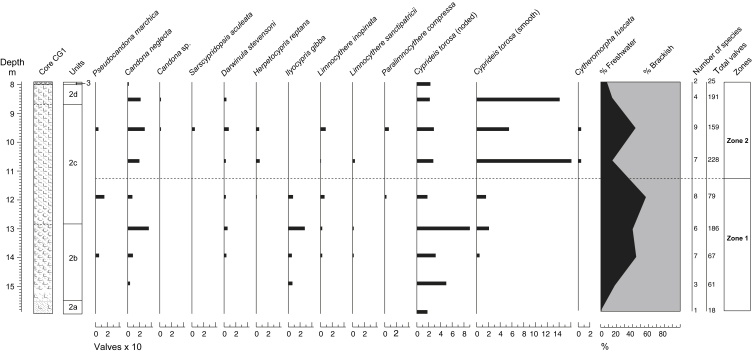
Ostracod diagram for core CG1. Nomenclature follows Meisch (2000).

Ostracods were recovered from the silty clays (Unit 2) and detritus muds (Unit 3) penetrated in borehole CG1. Depths given below refer to this borehole. The organic clays (Unit 4) and the basal sands and gravels (Unit 1) were barren. After wet-sieving, all identifiable fragments retained on 125 μm sieves were counted. Two local assemblage biozones, 1 and 2 were identified in the ostracod diagram (Fig. 9).

### 9.2 Assemblages

#### Biozone 1: 15.87–11.25 m

This lowermost zone extends from the sandy clays to the shelly silts (sub-units 2a–2c). The sandy clays below 14.00 m yielded a sparse ostracod fauna dominated by juveniles of the euryhaline species *Cyprideis torosa* (Jones). Many of the specimens of *C. torosa* showed signs of nodal development, a characteristic that has been attributed to salinities (<6‰) at the lowest limit of the species' range (cf. Meisch, 2000). Freshwater species increase in frequency and become more diverse in the clay-rich sediments at 13.00 m. These include the bottom-burrowing *Candona neglecta* Sars and *Pseudocandona marchica* Hartwig and the plant-crawling *Ilyocypris gibba* (Ramdohr) and *Limnocythere inopinata* (Baird). Two of these, *C. neglecta* and *I. gibba*, show particularly large increases in abundance at 13.00 m (Fig. 9). Noded valves of *C. torosa* also increase at this level.

#### Biozone 2: 11.25–7.90 m

This upper zone extends from the middle of sub-unit 2c to Unit 3 and is characterized by fluctuations in the frequencies of both brackish and freshwater taxa. At 10.63 m, for example, numbers of smooth-valved *C. torosa* increase dramatically, whilst the diversity and frequency of freshwater species decline. Numbers of *C. torosa* fall, however, at 9.50 m, with a corresponding rise in the frequencies of freshwater taxa. *C. neglecta* dominates the latter, with well-balanced numbers of adults and juveniles and males and females (Roe, 1994). The free-swimming *Sarscypridopsis aculeata* (Lilljeborg) also occurs. *C. torosa* valve frequencies rise again near the upper part of the unit. The thin detritus muds overlying the silty clays (Unit 3) yielded only a few noded *C. torosa* and the occasional broken valve of *C. neglecta*.

### 9.3 Local environment inferred from the Ostracoda

The mixed ostracod faunas are typical of a highly transitional inner estuarine environment in which the euryhaline *C. torosa* was able to flourish locally alongside fluctuating numbers of freshwater species. The early phase of silty clay deposition (sub-unit 2a) was characterized by conditions poorly suited to ostracod life, probably resulting from the sandy nature of the substrate or current activity. The presence of large numbers of noded *Cyprideis* suggests a salinity at ca 5‰ or less, if they are *in situ*.

In contrast, conditions during the accumulation of the overlying clay and silt dominated beds (sub-units 2b–2d) allowed *C. torosa* to thrive. Salinities were also low enough locally to enable *C. neglecta* and *I. gibba* to survive at all levels of development (Roe, 1994), the latter suggesting that aquatic or bankside vegetation existed within or in proximity to the channel (Meisch, 2000).

Salinities must have increased subsequently, as reflected by an expansion in smooth-valved *C. torosa* and a fall in local freshwater populations. Some of the sparse freshwater species were probably transported from sites further upstream. The well-balanced freshwater populations from the overlying sediments at 9.50 m appear to mark a brief decline in saltwater influence. Salinity levels increased again when the uppermost part of the silty clays accumulated.

Overall, the fauna of the silty clays typifies an estuarine section of river, which gradually became more open to marine influence through time. The brief lowering of salinities that took place in the latter part of this episode (at 9.50 m) may reflect a short-lived increase in river discharge, a change in tidal or depositional regime or possibly a small fall in relative sea level. This parallels the changes noted in the diatom floras at the same level.

The limited fauna of the detritus muds (Unit 3), points to a marked change in conditions, which were generally unfavourable for ostracods. The currents which carried sand and shell debris into the site at this time may have restricted their survival. Re-working from the lower silty clays also cannot be excluded.

## 10 Mollusca

### 10.1 Sampling and preparation

Small samples for molluscan analysis were taken at regular intervals throughout borehole CG1. Units 1, 2a, 4, 5 and 6 were barren but molluscan assemblages were recovered from sub-units 2c and 2d and Unit 3, whereas a few comminuted fragments were recovered from sub-unit 2b. The detritus muds (Unit 3) were easily the most shelly horizon. Consequently, the residues of large bulk samples from Unit 3 previously analysed for vertebrate remains by John Clayden (see Holman et al., 1990), were sorted and the frequencies of molluscan taxa estimated (Table 3).

### 10.2 Assemblages and environmental interpretations

The assemblages from the silty clays (sub-units 2c–2d) are dominated by brackish species, including *Hydrobia acuta* (=*Heleobia* cf. *neglecta*), a hydrobiid previously listed as ‘*Paladilhia radigueli*’, and the cockle *Cerastoderma glaucum*. Attribution of these hydrobiids to *H. acuta* is tentative but they possess tall slender shells with deep sutures that match reference material. *Peringia ulvae*, another hydrobiid tolerant of higher salinities, also occurs occasionally. This fauna is consistent with a quiet estuarine environment, which was sheltered from the influence of strong tidal activity. A brackish fluviatile setting appears more likely than a lagoonal or saltmarsh habitat, an interpretation that is further supported by the occasional presence of the freshwater gastropod *Valvata piscinalis*.

The faunas of the detritus muds (Unit 3) are more difficult to interpret because of the highly broken and probably allochthonous nature of the shells. Fourteen taxa have been recovered (Table 3). The preservation is generally poor and many of the shells are corroded. *H. acuta* and *C. glaucum* dominate the assemblage but freshwater species are also common, especially *Bithynia tentaculata*, *V. piscinalis*, *Ancylus fluviatilis*, *Corbicula fluminalis*, *Pisidium amnicum*, *Pisidium casertanum* f. *ponderosa* and *Pisidium henslowanum*/*supinum*. Other species include *Borysthenia naticina*, *Radix balthica* (=*Lymnaea peregra*) and *Pisidium clessini*. Fragments of freshwater mussel (*Anodonta* sp.) have also been recorded (Bridgland et al., 1988). No land snails were recovered. This fauna suggests an episode of inwashing from a nearby freshwater source, an event characterized by a marked change in depositional energies.

## 11 Coleoptera

### 11.1 Introduction

Samples taken throughout the organic clays (Unit 4) mostly yielded sparse, fragmentary and indeterminable insect remains. However, bulk samples (∼5 kg wet weight) from the detritus muds (Unit 3) exposed in a foreshore trench (Fig. 3) produced fossil Coleoptera that were well enough preserved to enable some taxa to be identified. The insect remains were recovered from the sediment using the method outlined by Coope (1986). Altogether 65 coleopteran taxa were recognised of which 47 could be determined to species level (Table 4). Seven taxa no longer live in the British Isles.

### 11.2 Environmental implications of the coleopteran assemblage

The large numbers of species represented by only one individual indicates that the assemblage is only a small subsample of the contemporary beetle fauna. The ecologically heterogeneous assemblage suggests that it was probably brought together in flood debris, some of it swept off the adjacent land surface, and some originating from a slowly moving river. This faunal variety means that the various ecological requirements of the species can be built up into a mosaic picture of the local environment.

#### 11.2.1 Aquatic habitats

*Oulimnius tuberculatus*, represented by a single specimen, is the only beetle indicative of running water, where it lives amongst stones and moss in shallow well-aerated riffles (Holland, 1972). However, the caddisfly *Hydropsyche contubernalis* McL. is usually found in large slow-flowing rivers (Edington and Hildrew, 1995). All the rest of the aquatic beetles are typical of standing or very slowly moving. The large carnivorous dytiscid taxa *Agabus bipustulatus*, *Ilybius* sp., *Acilius* sp. and *Dytiscus* sp. live in well-vegetated ponds. The hydrophilids *Coelostoma orbiculare*, *Cercyon convexiusculus* and *Hydrobius fuscipes* occur in similar habitats where they feed on decomposing plant material. *Chaetarthria seminulum* lives in wet mud, usually beside eutrophic pools or on the muddy banks of slow-flowing streams (Hansen, 1987). *Macroplea appendiculata* lives permanently underwater feeding mostly on *Potamogeton* but also on *Myriophyllum spicatum* and other pond-weeds. *Macronychus quadrituberculatus* lives in the fissures of submerged logs or rocks in fairly large, slow-moving rivers, where it probably feeds on algae. It is an extremely rare species in Britain today probably due to the reduction of its habitat by riverine management activities. *Tanysphyrus lemnae* is a minute weevil that feeds on the duckweed *Lemna* on the surface of stagnant water.

#### 11.2.2 Marginal damp habitats

In this category are included marsh habitats of varying degrees of humidity. By far the largest number of beetle species in this fauna fall into this group.

The Carabidae are ground beetles that are either predators or general scavengers. All the recovered species live close to still or slowly flowing eutrophic water where the soil is rich in humus and where the vegetation is rather lush and composed of *Carex*, *Scirpus* or *Phragmites*. *Bembidion unicolor* occurs in deciduous forests and is especially characteristic of the drier parts of *Alnus* swamps. Some species, such as *Loricera pilicornis* and *Platynus ruficornis*, require bare patches of soil. Of particular interest are the two carabids *Oodes gracilis* and *Odacantha melanura*, which inhabit accumulations of dead reeds, such as *Phragmites*, on the swampy borders of eutrophic lakes (Lindroth, 1992).

*Plateumaris braccata*, monophagous on *Phragmites communis* (Koch, 1992), was represented by numerous fragments. *Donacia cinerea* feeds on reeds and sedges, such as *Typha*, *Phragmites* and *Carex*. *Donacia semicuprea* and *Notaris acridulus* feed principally on the aquatic grass *Glyceria*. The weevil *Limnobaris pilistriata* chiefly feeds on Cyperaceae and similar plants; the larvae usually eating the roots. *Phalacrus caricis* feeds on the smutted inflorescences of various species of *Carex* (Thompson, 1958). *Dascillus cervinus* lives in damp meadows where the larvae feed on the roots of grasses.

#### 11.2.3 Meadow-like habitats

Hardly any xerophilous species were recovered except for the ‘click beetle’ *Adelocera murina*, which is common today in dry grassland where its larvae feed on the roots of various plants.

#### 11.2.4 Woodland habitats

The Coleoptera provide abundant evidence for the local presence of trees both living and dead. The Elm bark beetle *Scolytus scolytus*, the vector for Dutch Elm disease, is not restricted to *Ulmus* but also attacks *Fraxinus*, *Carpinus* and other deciduous trees. *Hylesinus crenatus* chiefly attacks *Fraxinus* and only exceptionally other broad-leaved trees. Both the adults and larvae of *Agelastica alni* (alder leaf beetle) eat the leaves of *Alnus*. The larvae of the weevil *Rhynchaenus testaceus* mine its leaves. *Stenoscelis submuricatus*, the commonest species in this assemblage, bores into the wood of diseased broad-leaved trees. *Rhyncolus elongatus* feeds in the trunks and stumps of partly rotting conifers, both *Pinus* and *Abies*, whereas *Hylobius abietis* (large pine weevil) attacks both pines and spruces.

Evidence of dead or dying trees is provided by a number of species. *Gastrallus immarginatus* burrows under dead bark of old oak trees but only rarely other deciduous trees. *Rhysodes sulcatus* lives in decaying wood, both deciduous and conifer, mainly in fallen trunks in moist places (Lindroth, 1986). It is extremely rare in Europe having a relict distribution from the times when the old natural forests were more widespread. *Valgus hemipterus*, *Osmoderma eremita* (hermit beetle) and *Dorcus parallelopipedus* (lesser stag beetle) develop in decaying wood of deciduous trees. *Prionus coriarius* is a large beetle (up to 45 mm) that lives in rotten trunks and major branches of various broad-leaved trees, less often on conifers; its larvae mostly feed on tree roots. *Anobium punctatum* (woodworm beetle) attacks dead wood that is moderately dry but which has often already been attacked by decomposing fungae (Hickin, 1963).

#### 11.2.5 Specialist habitats

A number of species occur with other specialised habitats. Thus *Onthophagus* and *Aphodius* are dung feeders. Species of *Hister* are most often found in carcasses where the larvae live on maggots. *Silpha tristis* is another carrion beetle that can feed on other dead insects (Koch, 1989). Their presence indicates that both dung and carcases must have existed on dry ground rather than in the marsh itself. *Phosphuga atrata* is a specialist predator on snails and slugs.

#### 11.2.6 Summary

This beetle assemblage indicates a sluggish river surrounded by a marsh dominated by *Phragmites*. Alder trees grew alongside the river. Nearby the mature forest consisted mainly of deciduous trees, including oak and ash, as well as coniferous trees such as pine. There is much evidence of dead and rotting timber. Some of the trunks were submerged in water. The presence of beetles that feed on dung and carrion indicates that the area was visited by large herbivorous mammals, perhaps attracted by the presence of *Glyceria* (sweet grass). There is no evidence of saline conditions, as would be indicated by obligate halobiont species.

### 11.3 Climatic inferences of the Coleoptera

This coleopteran assemblage is entirely composed of temperate species some of which have present-day geographical ranges that do not extend as far north as Britain. Of particular importance is the presence of *O. gracilis*, which has a strong preference for habitats that heat up rapidly in the summer (Lindroth, 1943). Several of the wood-dependent species (e.g. *R. sulcatus*, *V. hemipterus*, *R. elongatus* and *S. submuricatus*) are also predominantly ‘southern’ but these inhabitants of old mature forests may now be restricted by anthropogenic reduction of their habitats rather than by climate. However, there is no doubt that this fauna indicates a climate rather warmer than that in southern England today.

Using the Mutual Climatic Range (MCR) method (Atkinson et al., 1987) it is possible to give quantitative estimates of the thermal climate based on the coleopteran assemblage. Fourteen species in the Cudmore Grove fauna were also present on the MCR database (see Table 4). This method gave the following estimates, where *T*_max_ is the mean temperature of the warmest month (July) and *T*_min_ is the mean temperature of the coldest month(s), namely January and February:

*T*_max_ lay somewhere between 16 °C and 22 °C
*T*_min_ lay somewhere between −7 °C and 4 °C

Within this range of temperatures it is possible to give a best estimate (Coope et al., 1998). This gives the following figures for the most likely values:

$$T_{\max}=19\;{}^{\circ}\text{C,}\;T_{\min}=1\;{}^{\circ}\text{C}$$

Thus the mean July temperatures were about 2 °C warmer than those of the present day in southeast England and mean January temperatures may have been very slightly colder.

## 12 Vertebrates

### 12.1 Introduction

Fossiliferous deposits were first reported at East Mersea in the early 1900s by Dalton (1908), although no specific mention was made of vertebrate remains. Later accounts by Warren (1917, 1933) and Cornwall (1958) mention bones of *Hippopotamus* and therefore probably refer to the deposits of Ipswichian age that are known from the nearby ‘Hippopotamus Site’ (Bridgland and Sutcliffe, 1995) and the ‘East Mersea Restaurant Site’ (Bridgland et al., 1995) a short distance away on the foreshore (Fig. 1). The detritus muds (Unit 3) at Cudmore Grove have yielded an extensive vertebrate assemblage including mammals, birds and herpetiles (Bridgland et al., 1988; Holman et al., 1990) and fish. Unit 1 has yielded a fragmentary molar of an indeterminate elephant (G.R. Ward, personal communication) and a single bone of *Bison* or *Bos* was recovered from Unit 2 (Roe, 1994). The specimens from Unit 3 are extremely well preserved, showing no significant signs of rolling or abrasion. The presence of fragile herpetile vertebrae and small mammal mandibles with the dentition still *in situ* suggests that there has been little post-depositional disturbance.

At least 18 mammalian taxa have been recognized amongst the 1508 bones and teeth recovered from the detritus muds (Unit 3) (Table 5). In addition, 11 fish taxa, 14 herpetofaunal taxa (364 specimens) and 11 avian taxa (41 specimens) have been recorded.

### 12.2 Climatic and environmental interpretation of the vertebrate remains

#### 12.2.1 Fish

The 11 fish taxa recovered from Unit 3 belong to seven different families (Table 5). All are typical of freshwater rivers and streams, although a few can tolerate more saline habitats. The fish remains would therefore seem to have been transported from purely freshwater habitats upstream of tidal influence. The most interesting species is sturgeon (*Acipenser* sp.) represented by distinctive fragments of scute, the presence of which indicates that Unit 3 includes material transported by a sizeable stream or river. Interestingly, sturgeon (*Acipenser sturio*) was recovered from the interglacial beds at Purfleet in the Lower Thames region (Schreve et al., 2002; Fig. 2), a site which is believed to occupy a similar position in the Thames terrace sequence to Cudmore Grove (Bridgland, 1994; Schreve et al., 2002).

#### 12.2.2 Herpetofauna

The herpetofaunal assemblage, one of the richest reported from Britain (Holman et al., 1990), provides valuable palaeoclimatic information, since the geographical distributions of these animals are closely linked to temperature. All herpetiles are directly reliant upon external heat for their survival, and in the case of oviparous reptiles, require certain minimum temperatures for the hatching of their eggs. Six of the 14 herpetile taxa reported from Cudmore Grove by Holman et al. (1990) are not native in Britain: water frog or edible frog (*Rana ridibunda* or *Rana esculenta*), moor frog (*Rana arvalis*) tree frog (*Hyla* sp.), viperine or dice snake (*Natrix tessellata* or *Natrix maura*), aesculapian snake (*Zamenis longissimus*) and European pond terrapin (*Emys orbicularis*). There is some debate over the degree of oceanicity or continentality indicated by these exotic taxa. Based upon their present-day ranges, Holman et al. (1990) suggested that the palaeoclimate at Cudmore Grove was slightly warmer than at present in Britain, with mean July temperatures above 17–18 °C and with mild winters. Comparable modern analogues have been suggested for central or southern France (Holman et al., 1990) or northern Italy (Holman, 1993) but Gleed-Owen (1999) proposed more continental conditions. *Z. longissimus* and *N. tessellata* are distributed throughout most of eastern Europe, where they experience cold continental winters (Gasc et al., 1997). Taken in association with the occurrence of *R. arvalis* at Cudmore Grove, Gleed-Owen (1999) suggested that a more realistic modern analogue would be around the southeast German border with north-east Austria and the western Czech Republic.

The Cudmore Grove herpetofaunal assemblage is indicative of still or slowly flowing water with abundant aquatic vegetation, adjacent damp grassland and nearby well-vegetated, damp habitats with plenty of ground cover. Some drier, open shrub or woodland habitats are also indicated by *Bufo bufo* and *Z. longissimus*, whereas *Vipera berus* and *Lacerta vivipara* occur in a wide range of open terrestrial habitats, including heaths, dunes, woodland, marshes and meadowland (Arnold and Burton, 1980).

#### 12.2.3 Birds

Avian remains, which are usually under-represented in open sites, are represented by 42 identifiable bones or bone fragments. The presence of garganey (*Anas querquedula*), gadwall (*Anas strepera*) and little crake (*Porzana parva*) suggests summers as warm as that of central Europe at the present-day, in which case the northern boreal breeding whooper swan (*Cygnus cygnus*) and smew (*Mergellus albellus*) would probably have been present as winter visitors. These species suggest the presence of an open body of freshwater, with a well-vegetated margin and shallow-water plantlife. A range of passerines, including song thrush (*Turdus philomelos*), fieldfare (*Turdus pilaris*), great tit (*Parus major*), blue or coal tit (*Parus caeruleus* or *Parus ater*) and willow warbler or chiffchaff (*Phylloscopus trochilus* or *Phylloscopus collybita*) indicate the proximity of woodland. The Sandwich tern (*Sterna sandvicensis*) suggests the proximity of the coast.

#### 12.2.4 Mammals

The mammalian remains suggest a similar range of environments, with woodland and aquatic species particularly well represented. The presence of deciduous or mixed woodland with thick ground cover is suggested by the abundance of bank vole (*Clethrionomys glareolus*) and wood mouse (*Apodemus sylvaticus*), with smaller numbers of pygmy shrew (*Sorex minutus*) and serotine bat (*Eptesicus serotinus*), for which there are few British Pleistocene records. This is supported by larger mammal taxa such as red squirrel (*Sciurus vulgaris*), macaque (*Macaca sylvanus*), beaver (*Castor fiber*), brown bear (*Ursus arctos*), badger (*Meles meles*) and roe deer (*Capreolus capreolus*). Locally open vegetation is indicated by field vole (*Microtus agrestis*) and horse (*Equus ferus*), whereas aquatic habitats are indicated by the abundance of water vole (*Arvicola terrestris cantiana*), which represents 37% of the mammalian assemblage (MNI = 60), and the high frequency of water shrew (*Neomys* cf. *browni*), which frequent the well-vegetated banks of rivers and lakes with still or slow-flowing water. The presence of bicoloured white-toothed shrew (*Crocidura* cf. *leucodon*), which today has a predominantly southern European distribution, supports the inference that the climate may have been slightly warmer than at present.

## 13 Faunal biostratigraphy

The faunal datasets provide a wealth of information about the local environment and climate associated with the accumulation of the Cudmore Grove interglacial beds, but few groups are biostratigraphically significant. Neither the Coleoptera nor the Ostracoda provide definitive stratigraphical information. Amongst the Mollusca, however, the occurrence of *B. naticina* and *C. fluminalis* is significant, since neither species has been reported in the Lower Gravel or Lower Loam at Swanscombe in Kent (Fig. 1) or in the Freshwater Beds at Clacton. These horizons are thought to relate to the early-temperate sub-stage of the Hoxnian (Kerney, 1971; Turner and Kerney, 1971). During this interglacial in Britain *Corbicula* is known only from the late-temperate sub-stage, where it occurs in the Middle Gravel at Swanscombe, the Estuarine Beds at Clacton and in tidally-influenced sediments at East Hyde near Tillingham (Roe and Preece, 1995; Meijer and Preece, 2000; Roe, 2001). *B. naticina* is similarly known only from the upper levels (Middle Gravel and equivalents) at Swanscombe (Kerney, 1971). The occurrence of these species in brackish-water sediments belonging to a late-temperate sub-stage at Cudmore Grove would therefore be consistent with deposition during the Hoxnian. Other evidence presented in this paper suggests, however, that the organic sediments at Cudmore Grove belong to a later temperate stage, probably equivalent to MIS 9 (see below). Sites that have been correlated with this stage, such as Barling in Essex and Hackney Downs in central London, have yielded *Corbicula* during the early-temperate sub-stage (Bridgland et al., 2001; Green et al., 2006). It would appear that *Corbicula* occurred throughout much of MIS 9, in contrast to its more restricted occurrence during MIS 11. In Britain, *B. naticina* has not previously been recorded after the Hoxnian, and thus its occurrence at Cudmore Grove represents its first record from the late-temperate sub-stage of the ensuing interglacial.

The best faunal evidence for the age of the interglacial beds comes from the mammal remains. The assemblage lacks any of the ‘indicator species’ that have been found to characterise the Hoxnian (Schreve, 1997, 2001; Preece et al., 2007) at sites such as Hoxne (Stuart et al., 1993), Barnham (Parfitt, 1998), Beeches Pit, West Stow (Preece et al., 2007), all in Suffolk, Swanscombe (Sutcliffe, 1964; Schreve, 1996) and Clacton in Essex (Singer et al., 1973). Thus, in the case of the small mammals, the extinct small mole (*Talpa minor*), rabbit (*Oryctolagus cuniculus*), extinct giant beaver (*Trogontherium cuvieri*) and European pine vole (*Microtus* (*Terricola*) *subterraneus*) are all absent at Cudmore Grove, despite extensive sieving that led to the recovery of over 1500 mammalian remains. In Britain this assemblage of species only co-existed during the Hoxnian so their absence at Cudmore Grove strongly suggests that this site dates from a younger temperate episode. This is supported in the large mammal assemblage by the occurrence at Cudmore Grove of brown bear (*U. arctos*), as opposed to cave bear (*Ursus spelaeus*) known from the Hoxnian.

The distinction first made by Currant (1989) between the vertebrate assemblages from Hoxnian sites and those from Grays and Cudmore Grove was initially dismissed by Bridgland (1994), who suggested that variations in sample size might explain the apparent differences. However, Schreve (1997) counted 1575 specimens from 27 taxa at Grays, 1622 specimens from 33 taxa at Swanscombe and 952 specimens from 19 taxa at Clacton, compared with 1508 specimens from 18 taxa at Cudmore Grove. These counts demonstrate that all four sites are comparably rich, indicating that the absence of the Hoxnian ‘indicator species’ at Cudmore Grove is biostratigraphically significant, although it is true that the number of large mammals recovered from the other sites is far larger than the comparable total from Cudmore Grove.

On the basis of mammalian biostratigraphical evidence, in particular the presence of white-toothed shrews (*Crocidura* spp.), the Cudmore Grove deposits were originally grouped not only with those of Grays Thurrock (Essex) but also with the lower part of the sequence at Aveley (Essex) and Itteringham (Norfolk) (Bridgland et al., 1988; Currant, 1989). However, *Crocidura* has now been recovered from deposits of Hoxnian age at Barnham (Parfitt, 1998) and of Last (Ipswichian) Interglacial age at Tornewton Cave, Devon (A.P. Currant, personal communication) so, pending further refinement to species level, the genus can no longer be used as an indicator of age.

The Cudmore Grove mammalian assemblage compares most closely with those faunas known from Purfleet and Grays Thurrock in the Lower Thames region (Fig. 2). These localities have previously been attributed to a wide range of ages but have most recently been assigned to MIS 9 on the basis of terrace stratigraphy (Bridgland, 1994) and mammalian biostratigraphy (Schreve, 1997, 2001; Schreve et al., 2002). These assemblages are unified not only by the species present but also characterized by some that are absent, where such absence is not related to collection failure. Moreover, all three sites contain remains of water shrew, attributed to *N.* cf. *browni* on the basis of size. *N. browni* was first described by Hinton (1911) from Grays Thurrock and was considered by him to represent a transitional form between the small early Middle Pleistocene water shrew, *Neomys newtoni* and the larger, modern species *Neomys fodiens*. Whether this represents a valid species is open to question but relative size may nevertheless serve as a useful biostratigraphic marker (Schreve, 2001).

The remains of water vole (*A. terrestris cantiana*) from Cudmore Grove are also of biostratigraphical significance, since they show differences from those recovered from Hoxnian sites. *A. terrestris cantiana* is characterized by relatively small and permanently growing rootless molars, which display a ‘*Mimomys*’ enamel differentiation (i.e. the enamel of the trailing (convex) edges is markedly thicker than that of the leading (concave) edges). The modern NW European *Arvicola terrestris terrestris*, on the other hand, has relatively larger molars that display a ‘*Microtus*’ enamel differentiation, with thicker enamel on the leading (concave) edges of the salient angles. Throughout the Middle and Late Pleistocene, an evolutionary trend is apparent in the water vole lineage as the size of the molars increases and the thickened enamel on the convex sides of the molars reduces (Sutcliffe and Kowalski, 1976; Heinrich, 1982, 1987; Stuart, 1982; van Kolfschoten, 1990; von Koenigswald and van Kolfschoten, 1996). A separation of the Cudmore Grove sample from that of Hoxnian sites has been demonstrated on the basis of this enamel differentiation, using a quotient (SDQ) to express the relative thickness of the enamel on the trailing and leading edges of the molars. The SDQ values for Hoxnian *Arvicola* samples of first lower molars fluctuate around 140 (Parfitt, 1998; Schreve, 2001; Preece et al., 2007, Fig. 27), whereas those from Cudmore Grove have a mean value of 133.36 (*n* = 48, range = 105–147), implying a younger age. The SDQ values for Cudmore Grove accord well with those from other sites attributed to MIS 9, such as Grays Thurrock and Purfleet (Schreve, 2001; Preece et al., 2007).

A further trend is also visible in *Arvicola*, namely the progressive loss of the ‘*Mimomys* fold’ in the first lower molar. The earliest forms of *Arvicola* from the end of the ‘Cromerian Complex’ retain the ‘*Mimomys* fold’ and this trait consistently occurs in up to one third of individuals from Hoxnian deposits (Parfitt, 1998; Preece et al., 2007, Fig. 27). In later interglacials, that number has dwindled or even disappeared. The evidence from Cudmore Grove is consistent with a post-Hoxnian age, since of the 112 first lower molars recovered, the ‘*Mimomys* fold’ is present in only 5 specimens (4.46% of the sample).

An upper limit for the age of the Cudmore Grove interglacial is suggested by the presence of *M. sylvanus*, which is unknown in Britain from deposits attributed to MIS 7 and later. The length of the Cudmore Grove water vole first lower molars may also be significant, since they are smaller (mean = 3.49 mm, *n* = 33) than samples from localities attributed to MIS 7, such as Pontnewydd Cave (Green, 1984) (3.69 mm, *n* = 3, Schreve, 1997) and to the Ipswichian (see Preece et al., 2007, Fig. 27 for details). The distinctive mammalian assemblage from Cudmore Grove therefore differs from those described from several Hoxnian sites that have produced reasonable samples of mammalian remains. The absence of critical Hoxnian ‘indicator species’, the presence of both brown bear and the occurrence of *Arvicola* molars with relatively lower SDQ values, all indicate a post-Hoxnian age for the Cudmore Grove interglacial.

## 14 Archaeology

At least three unstratified flint flakes have been found on the beach at Cudmore Grove towards the southern channel margin (G.R. Ward and P. Spencer, personal communication). All these finds probably originate from the feather-edge of the Cudmore Grove Channel Gravel (Unit 1), from which a single struck flake was recovered *in situ* (Bridgland, 1994) (Fig. 5). A scraper has also been found on the foreshore to the immediate east of the Cudmore Grove sea wall (TM 06891466; Figs. 3 and 4). The presence of iron-stained sand within cavities of the scraper suggests it may have been derived from the adjacent Upper sands and gravels (Unit 6). A broken hand-axe has also been recovered from a foreshore exposure of gravel near the ‘Hippopotamus Site’ (TM 06541424; P. Spencer, personal communication). Its relationship to the infill sequence at this site is uncertain.

## 15 Amino acid geochronology

### 15.1 Introduction

A new technique of amino acid analysis has been developed for geochronological purposes (Penkman, 2005; Penkman et al., 2007a, 2008a), combining a reverse-phase high-pressure liquid chromatography (RP-HPLC) method of analysis (Kaufman and Manley, 1998) with the isolation of an ‘intra-crystalline’ fraction of amino acids by bleach treatment (Sykes et al., 1995). This combination of techniques results in the analysis of D/L (dextro- and laevo-rotatory optical isomers) values of multiple amino acids from the chemically protected protein within the biomineral, thereby enabling both decreased sample sizes and increased reliability of the analysis.

Amino acid racemization (AAR) analyses were undertaken on shells and opercula from bulk samples from Unit 3: four shells of *B. tentaculata* (NEaar 0859, 0888–0890; published in Penkman et al., 2007a), four shells of *V. piscinalis* (NEaar 0855–0858; published in Penkman et al., 2007a) and four opercula of *B. tentaculata* (NEaar 0891–0894). Seven *B. tentaculata* opercula (NEaar 2407–8, 3728–3730, 3820–2821) have also been analysed from the neighbouring ‘East Mersea Restaurant Site’ (Sample 3) (Fig. 1); five previously published by Penkman et al. (2007b) (NEaar 3728–3730, 3820–3821) and two new analyses (NEaar 2407–8). All samples were prepared using the procedures of Penkman (2005) and Penkman et al. (2008a) to isolate the intra-crystalline protein by bleaching. Two subsamples were then taken from each shell; one fraction was directly demineralised and the free amino acids analysed (referred to as the ‘Free’ amino acids, FAA, F), and the second was treated to release the peptide-bound amino acids, thus yielding the ‘total’ amino acid concentration, referred to as the ‘Total Hydrolysable amino acid fraction’ (THAA, H*). Samples were analysed in duplicate by RP-HPLC.

**Fig. 10:**
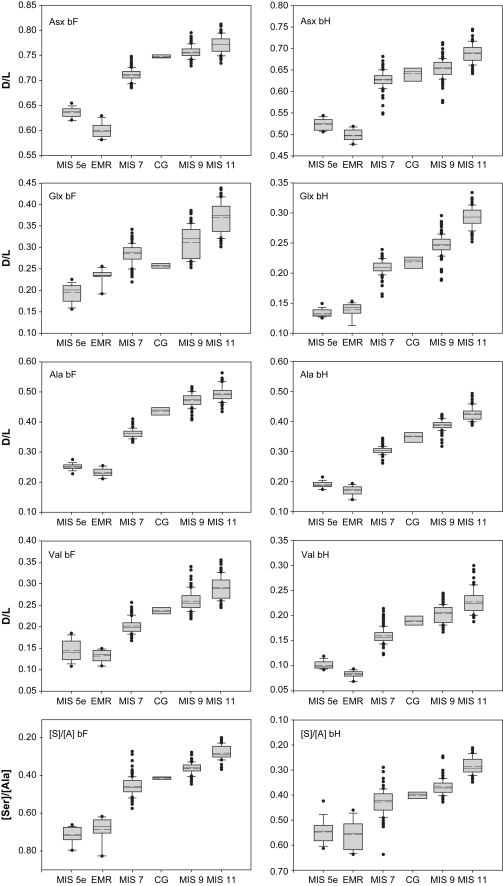
D/L values of Asx, Glx, Ala, Val; and [Ser]/[Ala] for the Free (FAA; F) and Total Hydrolysable amino acid (THAA; H) fractions of bleached *Bithynia tentaculata* opercula from East Mersea Restaurant Site (EMR) and Cudmore Grove (CG), compared with shells from sites correlated with MIS 5e (Bobbitshole, Trafalgar Square), MIS 7 (Aveley, Lion Pit), MIS 9 (Barling, Grays, Hackney, Purfleet) and MIS 11 (Swanscombe, Hoxne, Elveden, Barnham, Clacton and Beeches Pit). For each group, the base of the box indicates the 25th percentile. Within the box, the solid line plots the median and the dashed line shows the mean. The top of the box indicates the 75th percentile. Where more than 9 data points are available, the 10th and 90th percentiles can be calculated (shown by lines below and above the boxes respectively). The results of each duplicate analysis are included in order to provide a statistically significant sample size. The *y*-axes for the [Ser]/[Ala] data are plotted in reverse, so that the direction of increased protein degradation for each of the indicators remains the same. Note different scales on the *y*-axes.

The DL ratios of aspartic acid/asparagine, glutamic acid/glutamine, serine, alanine and valine (D/L Asx, Glx, Ser, Ala, Val) as well as the [Ser]/[Ala] value provide an overall estimate of protein decomposition (Fig. 10; Supplementary Online Appendix 1). Serine is one of the most geochemically unstable amino acids, with one of its decomposition products being alanine (Bada et al., 1978). This enables the ratio of the concentration of serine ([Ser]) to the concentration of alanine ([Ala]) to be used as a useful indication of the extent of protein decomposition. The D/L of an amino acid will increase with increasing time, whilst the [Ser]/[Ala] value will decrease. The D/L of Ser is less useful as a geochronological tool for samples of this age, but is presented here as aberrant values are useful indications of contamination.

### 15.2 Results

In a closed system, the amino acid ratios of the FAA and the THAA subsamples should be highly correlated, enabling the recognition of compromised samples. The extent of protein decomposition in both the FAA and THAA increases with time, with increased levels of protein breakdown during warm stages and decreased rates of degradation in cold stages. Data from British interglacial deposits using the intra-crystalline fraction of these three biominerals (*V. piscinalis* shell, *B. tentaculata* shell and opercula) tend to fall into clusters of amino acid ratios (Penkman, 2005; Penkman et al., 2007a). Given a similar temperature history, this then allows a regional aminostratigraphic framework to be developed, with independent geochronology allowing correlation with marine Oxygen Isotope Stages (Penkman, 2005). The extent of protein degradation in the *V. piscinalis* and *B. tentaculata* shells indicates an age for the Cudmore Grove shells between MIS 7 and MIS 11 (Penkman et al., 2007a).

It has become apparent that the protein fraction within gastropod shells has greater variability in samples of this age, resulting in difficulties in discrimination between different interglacial warm stages (Penkman et al., 2007a). Amino acid data obtained from the intra-crystalline fraction of the calcitic *Bithynia* opercula indicate that this biomineral is a particularly robust repository for the original protein (Penkman et al., 2007a, 2008a, in press). The *B. tentaculata* opercula from Cudmore Grove (Fig. 10) have protein that is significantly more degraded than that from Aveley and Lion Pit, which are in the Mucking Formation of the Thames, correlated with MIS 7 (Bridgland, 1994). Aveley also has OSL dates consistent with an age within MIS 7 (E. Rhodes, personal communication). The opercula protein from Cudmore Grove is also less degraded than that from sites correlated with MIS 11, such as Hoxne (Ashton et al., 2008), Swanscombe, Clacton (Bridgland et al., 1999), Barnham (Preece and Penkman, 2005), Elveden (Ashton et al., 2005) and Beeches Pit (Preece et al., 2007). Both Hoxne (Grün and Schwarcz, 2000) and Beeches Pit (Preece et al., 2007) have independent geochronology (U series/ESR and TL) confirming attribution to MIS 11. The opercula show similar levels of protein degradation to sites correlated with MIS 9, including Hackney (Green et al., 2006), Barling (Bridgland et al., 2001) Grays (Bridgland, 1994) and Purfleet (Schreve et al., 2002). Purfleet also has OSL data consistent with an MIS 9 age (E. Rhodes, personal communication). The intra-crystalline protein decomposition is slightly lower than that observed at Purfleet, which is consistent with an age within a later part of MIS 9.

Due to the low levels of degradation with cold stages and because of the natural variability in biological samples, there is a potential difficulty of discriminating between the end of one warm stage and the beginning of the next, particularly with some amino acids. We have therefore used statistical tests on the *B. tentaculata* opercula to establish whether it is possible to use the extent of protein breakdown to discriminate between Cudmore Grove and selected sites that have multiple lines of evidence correlating them with either MIS 11, MIS 9 or MIS 7. Together with the problem of small sample sizes, amino acid data have upper and lower limits (0 and 1) and therefore statistical tests must be applied with caution. However, they can provide useful insights alongside interpretations based on the graphical data alone. For each amino acid within each fraction (FAA and THAA), a 2-tailed *t*-test (assuming normally distributed data, as shown to be usual in Penkman et al., 2007a) was performed on the data. If the result of the *t*-test produces a *p* < 0.05, this enables discrimination between the two groups tested at a 95% confidence level. The 2-tailed *t*-test assumes no prior knowledge of the stratigraphy and therefore provides a useful guide of the resolving power of this technique when applied to sites of unknown age. The results are presented in Supplementary Online Appendix 2 but are also summarised in Table 6.

The amino acid data from *B. tentaculata* and *V. piscinalis* shells and *B. tentaculata* opercula indicate an age for Cudmore Grove lying between MIS 7 and MIS 11 but the enhanced chronological resolution from the *Bithynia* opercula data indicates a probable mid-late MIS 9 age.

The *B. tentaculata* opercula protein analysed from the East Mersea Restaurant Site (Sample 3) was significantly less degraded than those from Cudmore Grove (Fig. 10). Although one sample has slightly lower ratios in Glx and Val (NEaar 2408), the samples have very similar values to opercula from sites correlated with the Ipswichian (MIS 5e), such as Trafalgar Square in central London and Bobbitshole, the Ipswichian type site near Ipswich (Suffolk) (Penkman et al., 2008b). The extent of protein decomposition is significantly less than that observed in opercula from sites correlated with MIS 7 (Appendix 1). The amino acid data therefore support the correlation of the Restaurant Site with the Ipswichian.

## 16 Summary and discussion

### 16.1 Environment and climate associated with the interglacial beds: synthesis

The channel-fill sequence at Cudmore Grove has yielded an impressive suite of thermophilous plant and animal remains clearly indicative of interglacial conditions. Particularly important in this respect are the beetle assemblages, from which a temperature range *T*_max_ 16–22 °C and *T*_min_ −7 °C to +4 °C has been estimated, and the herpetofauna that is indicative of a climatic regime equivalent to that found today in parts of central Europe. Moreover, the Cudmore Grove channel sediments span much of the interglacial and have provided a long pollen record extending from the early-temperate stage, when oak and other thermophilous forest elements were expanding, to the late-temperate stage, when late immigrating trees such as *Abies* and *Carpinus* were becoming established (Table 7). The post-temperate sub-stage, during which pollen values of *Quercus*, *Carpinus* and *Abies* decline and those of *Pinus*, Poaceae and Ericales increase, may also be represented in the grey clays (Unit 5) in the upper part of the sequence.

Further insights into the depositional context of the interglacial beds have been provided by the diatom, ostracod, molluscan and coleopteran assemblages, which show that the channel-fill sequence accumulated in a dynamic coastal environment that underwent a number of changes through time (Table 7). The diatoms and ostracods together show that the silty clays (Unit 2) were deposited in a tidally-influenced river channel, which became increasingly open to marine influence as the oak-dominated early-temperate sub-stage progressed. Outer estuarine conditions prevailed by the end of this phase. Conditions changed markedly when the detritus muds (Unit 3) accumulated; this richly fossiliferous deposit was the product of high-energy riverine or coastal processes that carried shell debris, insect remains, bones and other detritus into the site from the surrounding hinterland and adjacent estuarine areas. Sheltered lagoonal conditions became established subsequently as the organic muds (Unit 4) accumulated and the site became isolated from the open estuary. The persistent presence of marine diatoms in these sediments, which date from the late-temperate sub-stage, confirms that relative sea levels remained high, with saltwater episodically penetrating the lagoon. The grey clays (Unit 5) capping the channel-fill sequence may even reflect a return to more open, estuarine or fully marine conditions towards the end of the interglacial, although the absence of diatoms or ostracods precludes confirmation.

### 16.2 The age of the Cudmore Grove interglacial beds

The biostratigraphical datasets have provided varying degrees of insight into the age of the interglacial beds. The pollen and plant macrofossil data are important not only for constraining the length of interglacial time recorded, but they additionally include elements that have traditionally been regarded as uniquely ‘Hoxnian’, including the presence of Type X pollen, modest levels of *Abies* and *Carpinus* pollen in the late-temperate sub-stage and megaspores of *A. filiculoides* (Turner, 1970; Phillips, 1976; Horton et al., 1992). A last interglacial (MIS 5e) or an MIS 7 age for the Cudmore Grove interglacial beds can therefore be firmly ruled out. However, given the uncertainty over the number and status of ‘Hoxnian-type’ interglacials represented in the British Pleistocene (Roe, 1994; Scourse et al., 1999; Thomas, 2001), there are potential flaws in undertaking correlations with spectra from other ‘Hoxnian’ sites. This problem is further compounded by the fact that most of the sites that have been attributed to the younger of the two ‘Hoxnian-type’ interglacials, which was possibly co-eval with MIS 9, have only yielded fragmentary pollen records, reflecting their fluvial origin (Thomas, 2001; Nitychhoruk et al., 2006). Notwithstanding this, the pollen spectra from Cudmore Grove zone CG-1 show some similarities to the short pollen profiles from Hackney (Green et al., 2006) and Barling (Bridgland et al., 2001) in the Thames system, which have both been correlated with MIS 9 on the basis of their faunal biostratigraphy and position in the Thames terrace sequence. These profiles, which span part of the early-temperate sub-stage of an interglacial (cf. Turner and West, 1968), also include a phase of *Quercus*–*Pinus* dominance with subsidiary levels of mixed oak forest taxa, e.g., *Ulmus* and *Fraxinus*. They have also both yielded macrofossils of *A. filiculoides*. Unfortunately, like many other early-temperate sub-stage spectra (cf. Turner and West, 1968), the observed palynological changes are biostratigraphically uninformative and do not provide independent grounds for correlation. Pollen biostratigraphical correlations with longer regional pollen records from lacustrine sequences are complicated by the fact that the Cudmore Grove pollen assemblages accumulated in a coastal environment where different taphonomic processes operate.

With regard to the molluscs, the occurrence of the invasive bivalve *C. fluminalis* in the early-temperate sub-stage is significant because during the Hoxnian it does not occur until the late-temperate sub-stage, at least in the Thames system (cf. Meijer and Preece, 2000). For example, at both Swanscombe and Clacton (Fig. 2), it only appears with the so-called ‘Rhenish fauna’ in the aggradations which are attributed to Ho IIIb (Kerney, 1971). Its early appearance at Cudmore Grove is again more consistent with its distribution at Barling (Bridgland et al., 2001) and Hackney Downs (Green et al., 2006) which both are attributed to MIS 9.

The mammalian faunas and the AAR data provide the strongest insights into the age of the Cudmore Grove interglacial beds and firmly support a correlation with MIS 9. In the case of the mammalian data, this correlation is not only based on the absence of several ‘indicator’ species of Hoxnian age, but on the presence of *U. arctos* (brown bear), which is unknown from the Hoxnian, and also on the ‘*Mimomys*’ enamel differentiation of the *Arvicola* molars (cf. Preece et al., 2007). The fact that only 4.46% of the *Arvicola* specimens from Cudmore Grove display the ‘*Mimomys* fold’ further points to a post-Hoxnian age (cf. Parfitt, 1998; Preece et al., 2007). The AAR datasets are derived from the shells of two species of molluscs (*V. piscinalis* and *B. tentaculata*), as well as the opercula of the latter. These data independently support a correlation with MIS 9. Despite their proximity and palynological similarities, the differences in the amino acid ratios between Cudmore Grove and Clacton are particularly striking. Not surprisingly, the Cudmore Grove amino acid ratios also differ from those of the East Mersea Restaurant Site (Bridgland et al., 1988, 1995), which lies only 2 km to the south-west of Cudmore Grove (Fig. 1). This interglacial deposit, which comprises ca 1.2 m of silts and gravel channelled into the London Clay, has yielded a freshwater molluscan fauna and a rich vertebrate assemblage (10 species) that includes the water vole, *A. terrestris terrestris* and *Hippopotamus amphibius*; a combination unknown in Britain before the Late Pleistocene (cf. Sutcliffe and Kowalski, 1976; Sutcliffe, 1995). The mammal assemblages compare closely with those from several other Ipswichian (MIS 5e) sites, including Joint Mintor Cave, Devon (Sutcliffe, 1960) and Trafalgar Square, London (Bridgland et al., 1995).

**Fig. 11:**
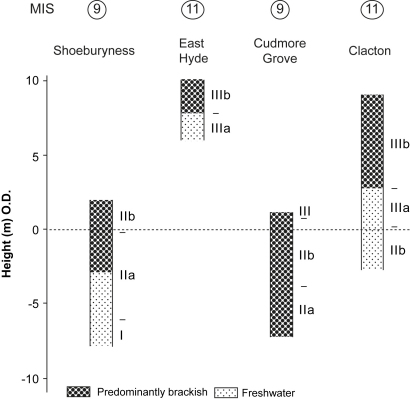
Altitudinal plot of interglacial channel-fill sediments in eastern Essex showing the salinity characteristics inferred from diatoms and ostracods and their associated pollen zones. The assemblage zones follow the terminology of Turner and West (1968). Early-temperate sub-stage = zone II; late-temperate sub-stage = zone III. The record from East Hyde follows Roe (2001); Clacton (Pike and Godwin, 1953; Turner and Kerney, 1971), Shoeburyness (Roe, 1999). The correlations proposed in the text with MIS 9 and 11 are indicated.

**Fig. 12:**
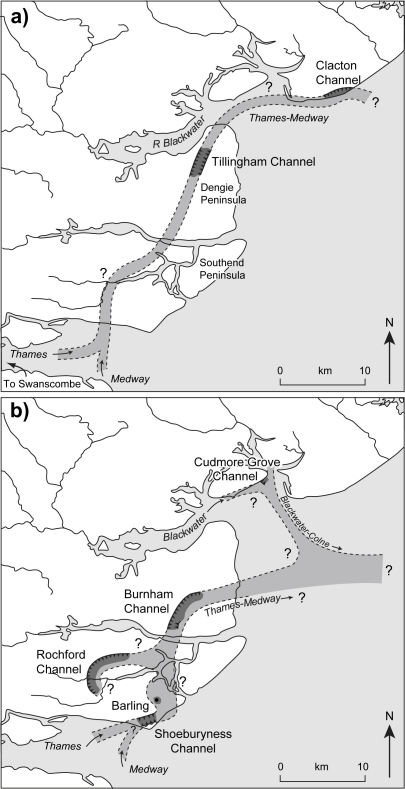
Palaeodrainage maps of eastern Essex during MIS 11 (a) and MIS 9 (b). Map a) shows the tidally-influenced Thames–Medway flowing between Tillingham (East Hyde) and Clacton during the late-temperate sub-stage of the inferred MIS 11 interglacial. The sequence at Swanscombe is considered to be the upstream equivalent of this channel system (Bridgland, 1994; Roe and Preece, 1995). Map b) depicts a larger, more complex palaeo-estuary inferred for the end of the early-temperate sub-stage of the younger interglacial, when lower estuarine conditions prevailed at Cudmore Grove (after Roe, 1994; Schreve et al., 2002). The Cudmore Grove palaeo-channel is placed in the more northerly part of this estuarine system and is assumed to have formed a tributary of the main Thames–Medway estuary. The dark shading shows the distribution of channel-fill sediments of estuarine character which have been examined for pollen and other microfossils (Roe, 1994).

Further independent support for a separation of the Cudmore Grove interglacial beds from those at Clacton is provided by the marked differences in the sea-level histories of the two sites. The Clacton Channel deposits do not record the onset of estuarine/marine conditions until relatively late in the interglacial, during the late-temperate sub-stage (Ho IIIb), whereas the earlier part of the channel-fill sequence, representing the early-temperate sub-stage, accumulated in a freshwater environment (Turner and Kerney, 1971; Bridgland et al., 1999). A similar onset of marine conditions, again during Ho IIIb, is recorded in the channel-fill sequences at East Hyde near Tillingham (Roe, 2001) (Fig. 11) and at Swanscombe (Kerney, 1971) and all three channels are believed to have infilled during MIS 11 (Bridgland et al., 1999; Roe, 2001; Fig. 12a). At Cudmore Grove, in contrast, estuarine conditions were already established in the early-temperate sub-stage of the interglacial represented, and relative sea levels remained high until at least the late-temperate sub-stage. It is difficult to reconcile the presence of outer estuarine conditions at Cudmore Grove during the early-temperate sub-stage with the freshwater conditions at Clacton; the channels lie at approximately the same height and are only 10 km apart and are thus likely to have been flooded by the sea around the same time had they formed during the same interglacial (Roe, 1994). To accommodate these differences within the same temperate event requires a complex model of coastal evolution, which would have allowed the sea to penetrate the eastern part of Mersea Island, but not neighbouring Clacton (Roe, 1999). Whilst this cannot be ruled out, it is not supported by any evidence or the bio- and aminostratigraphical data.

The clast lithological and palaeocurrent data presented here further re-enforce the interpretation that the Cudmore Grove Channel is not an upstream equivalent of the Clacton Channel as was once suggested (e.g. Bridgland, 1988, 1994). This interpretation was based on the assumption that both interglacial channel-fills were overlain by what were assumed to be Thames–Medway gravels: the Mersea Island Member aggradation above the Cudmore Grove Channel and the Wigborough Gravel at Clacton (Fig. 2). The fine-grained channel-fills were thus thought to represent the interglacial course of the Thames–Medway during the interglacial that preceded the Mersea Island–Wigborough Gravel aggradation: the Hoxnian (Bridgland, 1994, Fig. 5.5). The Hoxnian correlation was based on the biostratigraphy of the Clacton deposits and their position in the Thames terrace sequence (cf. Turner and Kerney, 1971; Bridgland, 1988, 1994, Fig. 5.5). The clast lithological data presented here cannot confirm that either the basal sands and gravels lining the Cudmore Grove Channel (Unit 1) or the Unit 6 gravels in the Cudmore Grove cliffs were deposited by the Thames–Medway. Indeed, the Unit 1 gravels have a composition that is more consistent with aggradation by a smaller river, possibly the Essex Blackwater, re-working Thames–Medway deposits, local Kesgrave Member gravels and fluvio-glacial gravels from the area to the west of Cudmore Grove. Moreover, the palaeocurrent directions from Unit 6 confirm that the upper part of this aggradation (sub-unit 6b) was deposited by a river flowing from the northeast (possibly a proto-River Colne), which is inconsistent with a Thames–Medway origin. The Unit 6 gravels also display compositional differences from Thames–Medway gravels further to the south, for example, on the Dengie Peninsula (cf. Bridgland, 1994), which suggest that they too may have been aggraded by tributary rivers (the Colne, Blackwater or both) re-working older Thames–Medway deposits. The terrace gravels above the two channels cannot therefore be used to confirm that the underlying interglacial beds are either contemporaneous or part of the same palaeo-channel system.

There is therefore compelling biostratigraphical, aminostratigraphical and palaeo-sea level evidence, together with evidence from the regional terrace stratigraphy to suggest that interglacial deposits from three separate interglacials are represented in this small area of eastern Essex: i) the Clacton and Tillingham Channel deposits, which are of Hoxnian age and formed during part of MIS 11 (Bridgland et al., 1999; Roe, 2001); ii) the Cudmore Grove Channel deposits, which we suggest formed within MIS 9; and iii) the East Mersea Restaurant Site deposits, which are of last interglacial (Ipswichian) age (MIS 5e). We reject the possibility that the Cudmore Grove sequence dates from a younger temperate sub-stage within MIS 11 (for example, sub-stage 11c) because the sequence records a full interglacial forest succession and temperatures several degrees warmer than present, characteristics that are inconsistent with other records attributed to this warm interval (cf. Reille et al., 2000; Desprat et al., 2005; Preece et al., 2007). The Cudmore Grove mammal faunas are also markedly different to those recorded in the upper part of the Hoxnian type-sequence at Hoxne (strata C and B), which has recently been re-assigned to a later sub-stage within MIS 11 (Ashton et al., 2008).

The palaeogeographies of eastern Essex were quite different during the two late Middle Pleistocene interglacials (Fig. 12). During MIS 11, the Thames–Medway appears to have flowed north-eastwards between Tillingham and Clacton, maintaining its early post-diversion route, which had been established late in the Anglian (cf. Bridgland, 1994; Fig. 12a). During MIS 9, a larger fluvio-estuarine drainage network appears to have existed in the region, of which the Cudmore Grove Channel sequence was only a small remnant (cf. Bridgland, 1994; Roe, 1994; Schreve et al., 2002) (Fig. 12b). On the basis of the clast lithological data, we assume that this section of the palaeo-estuary was associated with a southward-draining tributary system (the ‘proto-Colne or proto-Blackwater’), which may have become confluent with the Thames–Medway somewhere in the current offshore area, possibly east of the Dengie Peninsula. Interglacial deposits filling buried channels at Shoeburyness, Canewdon (Roe, 1999) and Barling (Bridgland et al., 2001) on the Southend Peninsula, and Burnham (North Wick) on the Dengie Peninsula (Roe, 1999) are all considered to have formed part of this palaeo-estuary (Roe, 1999; Schreve et al., 2002; Figs. 1 and 12). Like Cudmore Grove, all these sites have yielded brackish-water assemblages indicative of the early-temperate sub-stage of an interglacial (Roe, 1994, 1999). The spectra from Shoeburyness and North Wick show striking similarities to those from zone CG-2 at Cudmore Grove, although the late-temperate sub-stage is missing at these sites (Roe, 1999). The exact geography of this palaeo-estuary is speculative, although bathymetric and seismic data from the offshore area to the east of the Dengie Peninsula confirm the existence of a buried channel system with associated submerged terrace gravels in the postulated vicinity of this inferred MIS 9 estuary (Bridgland et al., 1993; Bridgland and D'Olier, 1995; Bridgland, 2002). It is worth noting that the occurrence of channel-fills in close proximity that can demonstrably be shown to date from different interglacials is not unique in Britain; a similar series of channel-fills of Middle to Late Pleistocene age occur in close juxtaposition in the eastern Solent on the south coast of England, incised into the soft, Eocene Bracklesham Beds (Preece et al., 1990).

### 16.3 Correlations with other regional sites of inferred MIS 9 age and wider significance

The data presented here form part of a growing body of evidence that supports the existence of a second interglacial in the British late Middle Pleistocene, which included some ‘Hoxnian’ palaeovegetation elements (cf. Scourse et al., 1999; Thomas, 2001; Scourse, 2006). This interglacial has been correlated with MIS 9 on the basis of the widely held assumption that the Anglian can be equated with MIS 12 and the Hoxnian with part of MIS 11 (cf. Bridgland, 1994; Rowe et al., 1999; Grün and Schwarcz, 2000; Schreve, 2001; Preece et al., 2007). In addition to Cudmore Grove and the aforementioned interglacial estuarine deposits in southern Essex (Shoeburyness, Barling, North Wick and Canewdon), the interglacial beds at Purfleet and Grays (Bridgland, 1994; Schreve et al., 2002; Fig. 2) and Hackney Downs (Green et al., 2006) have all been assigned to this temperate event. These deposits have been separated from the Hoxnian and later temperate stages using aminostratigraphy, mammalian biostratigraphy and by their position in the Thames terrace sequence; in no case has separation been possible on the basis of pollen biostratigraphy alone. Of these sites, the sequence at Cudmore Grove is by far the most complete, and, we argue, the most important: it spans a considerable part of the interglacial, provides detailed insights into the climate and local environment associated with this temperate stage, and evidence of high relative sea levels in the southern North Sea region. Moreover, the record shows that the marine transgression of this interglacial differed in its timing in this region from that of the preceding Hoxnian, taking place during the oak-dominated, early-temperate sub-stage. The maximum height attained by this inferred MIS 9 marine transgression is less easy to ascertain, as the Cudmore Grove interglacial beds are locally compacted and do not necessarily record the maximum flooding surface, which may or not be represented in the grey clays (Unit 5). Evidence from the interglacial channel-fills on the Southend Peninsula suggests that relative sea levels attained a height of at least ca 5–7 m O.D. in this region during the early-temperate sub-stage of this interglacial (Roe, 1999; Bridgland et al., 2001). This compares with the estimate of ca >20 m O.D. inferred for the Hoxnian RSL highstand during Ho IIIb from Swanscombe (Kerney, 1971; Bridgland, 1994). However, the possibility of differential post-depositional movements associated with long-term tectonic subsidence in the Thames Estuary (cf. D'Olier, 1972) means that comparisons of the highstand maxima must be made with caution.

Flint artefacts indicate that hominins were present during the infilling of the Cudmore Grove Channel, as they were at contemporary sites such as Grays and Purfleet in the Lower Thames (Schreve et al., 2002). A full archaeological excavation of the margins of the channel may therefore be worthwhile.

Perhaps the most important attribute of the Cudmore Grove record are the insights that it provides into the vegetation history of MIS 9 in southern Britain, particularly its record of the late-temperate sub-stage, which is missing from all the other regional sites presumed to be of this age (cf. Thomas, 2001). Interestingly, one possible exception is the interglacial sequence in the Nar Valley, Norfolk, which has long been correlated with the Hoxnian (e.g. Stevens, 1960; Ventris, 1986, 1996; Gibbard et al., 1992). Here, a complex succession of freshwater and marine deposits, locally over 10 m thick and extending up to 23 m O.D., overlies Anglian glacial sediments (Woodlands Farm Member of the Lowestoft Formation) (Ventris, 1986; Gibbard et al., 1992; Lewis, 1999). The pollen spectra from the marine beds include well defined *Abies*, *Carpinus* and *Empetrum* peaks, which have been ascribed to the Hoxnian late-temperate sub-stage (Stevens, 1960; Ventris, 1986, 1996), whereas the same unit has also yielded a diatom flora indicative of a nearshore, shelf environment (Mitlehner, 1992). This Hoxnian correlation has recently been challenged by ^230^Th/^238^U dates from peats within the Nar Valley freshwater beds, which have produced a mean age of 317 ± 14 ka, suggesting a correlation with MIS 9 (Rowe et al., 1997). Conventional isoleucine epimerization determinations on the foraminiferid *Ammonia beccarii* from the Nar Valley marine beds add independent support to this interpretation (Scourse et al., 1999). The mean aIle/Ile ratio obtained (0.135) is much lower than ratios (ca 0.2) based on the same species from the offshore interglacial Sand Hole Formation in the Inner Silver Pit, ca 20 km off the coast of East Yorkshire (Fig. 1). This deposit also yielded high levels of *Abies* and *Picea* and has similarly been ascribed to the Hoxnian late-temperate sub-stage (Scourse et al., 1998). These differences in ratios might reflect contrasting post-depositional thermal histories between the two sites, but it seems more likely that the Nar Valley and the Sand Hole Formation interglacial beds date from two temperate stages (MIS 9 and MIS 11 respectively) characterized by similar palaeovegetation (Scourse et al., 1999). If this interpretation is correct, then the widely held correlation of the Anglian glacial sediments in the Nar Valley with MIS 12 is thrown into question, although Scourse et al. (1999: p. 554) suggest that a substantial hiatus may exist between the Woodlands Farm Member and the Nar Valley freshwater beds. This warrants further investigation.

The stratigraphic separation of the Cudmore Grove interglacial deposits from those at neighbouring Clacton directly thus directly parallels the separation that has been made between the Nar Valley interglacial beds and the Sand Hole Formation on the basis of conventional aminostratigraphy and uranium series dating. Together these findings imply that: i) *Abies* was indeed a constituent of the regional forests in southern Britain during the late-temperate sub-stages of two successive late Middle Pleistocene interglacials, which are suggested to be equivalent to MIS 9 and 11; and ii) these interglacials were both characterized by high relative sea levels in the Southern North Sea region. This postulated distribution of *Abies* in the British late Middle Pleistocene tallies with the inferred distribution of the tree from the maar lake records of the Velay region, central France, where *Abies* appears to have been a significant forest component in both the latter stages of the Praclaux (MIS 11) and Landos (MIS 9) interglacials (Reille et al., 2000; de Beaulieu et al., 2001), although it was also present on a significantly reduced scale in the Bouchet 2 and 3 interstadials, which are correlated with MIS 7 (Reille et al., 2000). Such long-distance comparisons must be made with caution, however, as the composition of the interglacial forests of central France may have differed to those prevailing in southern Britain. Certainly the collective evidence from Britain implies that the biostratigraphical significance formerly attributed to *Abies* as a Hoxnian ‘indicator taxon’ in the late Middle Pleistocene should now be abandoned.

On a wider European scale, the evidence for two marine highstands associated with interglacial deposits of ‘Hoxnian-type’ character in the Southern North Sea region has more far-reaching implications, for it follows that the ‘Holsteinian’ marine beds of continental Europe may also record more than one interglacial high sea-level event. In short, we refute the contention that the issue of “two Holstein-like Interglacials” is “a problem confined to the Quaternary of the British Isles” as advocated by Geyh and Müller (2006: p. 3072). Whether polleniferous marine sediments of the inferred MIS 9 and MIS 11 highstands are widely present in other areas of the Southern North Sea and western Baltic regions remains to be seen; this paper has certainly highlighted their rather patchy distribution in southern Britain. What has become clear is the need to continue to adopt a multidisciplinary approach, supplementing pollen biostratigraphy with other more rigorous stratigraphical and geochronological techniques, to separate and better constrain these interglacials with ‘Hoxnian-like’ affinities.

## Figures and Tables

**Table 1:** Clast lithological composition of gravels from Mersea Island. Samples 1a and 1a are from Unit 1 (basal sands and gravels) sampled in borehole CG1 at Cudmore Grove; samples 6a–6c are from Unit 6 (upper sands and gravels) (Fig. 4). Other sample numbers follow Bridgland (1983, 1994).

				Local			Southern		Exotics						
Sub-division	Location	Sample no.	Height (m O.D.)	Non-Tertiary flint (%)	Tertiary flint (%)	Total (%)	Total greensand chert (%)	Total (%)	Quartz & quartzite (%)	Total (%)	Ratio Tertiary: non-Tertiary	Ratio Southern:local	Ratio Southern:exotics	Total count	Grid reference
Mersea Island Member	Cliff section	6a	5.1	54.8	33.3	88.1	7.3	7.3	4.6	4.6	1.65	12.07	0.63	562	TM 0683 1457
	Cudmore Grove														
	Cliff section	6b	6.3	57.8	28.3	86.1	10.3	10.3	3.6	3.6	2.04	8.35	0.35	417	TM 0683 1457
	Cudmore Grove														
	Cliff section	6c	7.8	57.3	31.7	89.0	8.5	8.5	2.2	2.2	1.80	10.39	0.28	409	TM 0683 1457
	Cudmore Grove														
	West Mersea^a^	1	19.3	43.8	38.6	82.4	14.2	14.2	2.8	3.5	1.13	5.80	0.24	578	TM 0134 1361
	West Mersea^a^	2	19.4	42.9	44.8	87.7	10.0	10.0	1.4	2.3	0.95	8.79	0.23	431	TM 0144 1373
	Fen Farm^a^	1	10.6	39.6	47.6	87.2	10.7	10.7	1.8	2.2	0.83	8.17	0.20	553	TM 0590 1444
	East Mersea														
	Fen Farm^a^	2	11.8	37.7	52.3	90.0	7.6	7.6	1.8	2.3	0.72	11.82	0.31	512	TM 0583 1437
	East Mersea														

Cudmore Grove Member	Borehole CG1	1a	−8.30 to −9.40	70.7	13.2	83.9	2.9	2.9	12.0	13.2	5.36	29.00	4.57	483	TM 0681 1456
	Cudmore Grove Channel Gravel														
	Borehole CG1	1b	−8.30 to −9.40	73.5	11.8	85.3	5.0	5.0	9.3	9.7	6.22	5.86	1.94	525	TM 0681 1456
	Cudmore Grove Channel Gravel														
	Cudmore Grove Channel lag gravel^b^	1	ca 1–2 m	40.1	44.9	85.0	14.0	14.0	1.0	1.0	1.12	6.07	0.07	301	TM 0682 1451

a Counts reported by Bridgland (1983).

b Counts reported by Bridgland (1994).

**Table 2:** Plant macrofossils preserved after the sieving stage of pollen analysis in samples from borehole CG1. None of the other pollen samples analysed from this core yielded identifiable macrofossils.

Pollen zone	Depth (m)	Unit	Terrestrial	Waterside & damp ground			Aquatic					Unclassified	
			*Urtica* sp(p). seeds	*Eupatorium cannabinum* L. achenes	*Typha* sp(p). fruit	*Alisma* sp. fruit	*Elatine hydropiper* L. fruit	*Ranunculus* Batrachian sp. achenes	cf. *Chara* sp(p). oospores	*Azolla filiculoides* Lam. megaspores	*Najas minor* All. fruit	Bryophyte fragments	*Sphagnum* leaves
CG-3	6.62	4	1										
CG-3	6.82	4		1									
CG-3	6.94	4			2				2			1	1
CG-3	7.02	4		1						3	1		
CG-3	7.12	4	1	1	1				1				
CG-3	7.21	4								3	1		
CG-3	7.31	4							1	5			1
CG-3	7.40	4								1			
CG-3	7.50	4			1	1			1				
CG-3	7.58	4				1							
CG-3	7.66	4			1								
CG-3	7.75	4	1										
CG-3	7.85	3			1							1	
CG-2	9.80	2c										1	
CG-2	12.20	2c			1								
CG-1	12.80	2b			1								
CG-1	13.00	2b			2								
CG-1	13.40	2b										1	
No pollen	15.10	2b			2								
No pollen	15.30	2a	1										1
No pollen	15.87	2a					1	1				1	
No pollen	16.00	2a					1						

**Table 3:** Estimated abundance of molluscan taxa in Units 3 and sub-units 2c and 2d. Abundant = >200, very common = 76–200, common = 21–75, occasional = 6–20, rare = 1–5.

	Sub-units 2c–2d	Unit 3
**Freshwater taxa**		
*Valvata piscinalis* (Müller)	Occasional	Common
*Borysthenia naticina* (Menke)	–	Occasional
*Bithynia tentaculata* (Linnaeus)	–	Very common
*Radix balthica* (Linnaeus) (=*Lymnaea peregra* (Müller))	–	Rare
*Ancylus fluviatilis* Müller	–	Common
*Anodonta* sp.	–	Rare
Unionidae	–	Occasional fragments
*Corbicula fluminalis* (Müller)	–	Abundant
*Pisidium amnicum* (Müller)	–	Common
*Pisidium clessini* Neumayr	–	Rare
*Pisidium casertanum* f. *ponderosa* Stelfox	–	Common
*Pisidium henslowanum* (Sheppard)/*P. supinum* Schmidt	–	Very common
*Pisidium subtruncatum* Malm	–	Rare

**Brackish-water taxa**		
*Hydrobia acuta* (Draparnaud) (=*Heleobia neglecta* (Muus))	Abundant	Abundant
*Peringia ulvae* (Pennant, 1777)	Rare	–
*Cerastoderma glaucum* (Poiret)	Common	Abundant
*Cerastoderma* sp. (probably *glaucum*)	Common fragments	Common

**Table 4:** Coleoptera recorded in a bulk sample of Unit 3 (detritus muds) collected from the foreshore trench section (Fig. 3). Nomenclature follows Lucht (1987).

**Carabidae**		**Helodidae**	
*Elaphrus cupreus* Duft^b^	1	Gen. et sp. indet	1
*Loricera pilicornis* (F.)^b^	1		
*Bembidion* (*Peryphus*) sp.	1	**Dryopidae**	
*Bembidion unicolor* Chaud.^b^	1	*Drypos* sp.	1
*Patrobus atrorufus* (Ström)^b^	1	*Oulimnius tuberculatus* (Müll.)	1
*Pterostichus strenuus* (Panz.)^b^	1	*Macronychus quadrituberculatus* Müll.	2
*Pterostichus nigrita* (Payk.)^b^	2		
*Pterostichus gracilis* (Dej.)^b^	1	**Phalacridae**	
*Platynus ruficornis* (Goeze)	1	*Phalacrus caricis* Sturm	1
*Oodes gracilis* Villaa, b	2		
*Odacantha melanura* (L.)^b^	1	**Anobiidae**	
		*Gastrallus immarginatus* (Müll.)	1
**Dytiscidae**		*Anobium punctatum* (Geer)	2
*Agabus bipustulatus* (L.)^b^	1		
*Ilybius* sp.	1	**Scarabaeidae**	
*Acilius* sp.	1	*Onthophagus* spp.	3
*Dytiscus* sp.	1	*Aphodius* sp.	1
		*Valgus hemipterus* (L.)a, b	1
**Rhysodidae**		*Triodonta* sp.^a^	1
*Rhysodes sulcatus* (F.)^a^	1	*Osmoderma eremita* (Scop.)^a^	1
			
**Hydraenidae**		**Lucanidae**	
*Hydraena* sp.	2	*Dorcus parallelopipedus* (L.)	1
*Ochthebius* sp.	1		
		**Cerambididae**	
**Hydrophilidae**		*Prionus coriarius* (L.)	2
*Coelostoma orbiculare* (F.)^b^	1		
*Cercyon convexiusculus* Steph.	2	**Chrysomelidae**	
*Hydrobius fuscipes* (L.)^b^	1	*Macroplea appendiculata* (Panz.)	2
*Chaetarthria seminulum* (Hbst.)^b^	1	*Donacia semicuprea* Panz.	1
		*Donacia cinerea* Hbst.	1
**Histeridae**		*Plateumaris sericea* (L.)	1
*Hister* (*sensu lato*) sp.	1	*Plateumaris braccata* (Scop.)	3
		*Agelastica alni* (L.)	1
**Silphidae**			
*Silpha tristis* III.	1	**Scolytidae**	
*Phosphuga atrata* (L.)	1	*Scolytus scolytus* (F.)	1
		*Hylesinus crenatus* (F.)	1
**Staphylinidae**			
*Aploderus caesus* (Er.)^a^	1	**Curculionidae**	
*Trogophloeus* sp.	1	*Apion* sp.	1
*Stenus* sp.	1	*Rhyncolus elongatus* (Gyll.)^a^	1
*Philonthus* sp.	1	*Stenoscelis* (*Brachytemnus*) *submuricatus* (Schoen.)^a^	4
		*Tanysphyrus lemnae* (Payk.)	1
**Pselaphidae**		*Notaris scirpi* (F.)	1
*Pselaphus heisei* Hbst.	1	*Notaris acridulus* (L.)	1
		*Anthonomus* sp.	1
**Elateridae**		*Hylobius abietis* (L.)	1
*Adelocera murina* (L.)	2	*Limnobaris pilistriata* (Steph.)	1
		*Phytobius* sp.	1
**Dascillidae**		*Rhynchaenus testaceus* (Müll.)	2
*Dascillus cervinus* (L.)	1		

a Species no longer living in Britain.

b Species present in the MCR database.

**Table 5:** Vertebrates recorded in a bulk sample of Unit 3 (detritus muds). (NIS = number of identified specimens, MNI = minimum number of individuals). Percentage values represent the abundance of each taxonomic group (e.g. Aves and Mammalia). Amphibia and Reptilia percentage totals are grouped and the identifications follow Holman et al. (1990).

Species	Present	Common	Abundant
PISCES			
**Acipenseridae**			
*Acipenser* sp., sturgeon	+		
**Salmonidae**			
*Salmo* sp., salmon or trout	+		
**Esocidae**			
*Esox lucius* L., pike			+++
**Cyprinidae**			
*Tinca tinca* L., tench	+		
*Abramis brama* L., common bream	+		
*Leuciscus leuciscus* L., dace	+		
*Rutilus rutilus* L., roach		++	
*Scardinius erythropthalmus* L., rudd	+		
**Anguillidae**			
*Anguilla anguilla* L., eel		++	
**Gasterosteidae**			
*Gasterosteus aculeatus* L., three-spined stickleback	+		
**Percidae**			
*Perca fluviatilis* L., perch	+		


Species	N.I.S.	% of Total within each group	M.N.I.
AMPHIBIA			
**Salamandridae**			
*Triturus cristatus* (Laurenti), warty newt	145	39.83	3
*Triturus vulgaris* L., smooth newt	2	0.54	1
*Triturus* sp., indeterminate newt	5	1.37	1
**Bufonidae**			
*Bufo bufo* L., common toad	12	3.29	6
**Hylidae**			
*Hyla* sp., tree frog	1	0.27	1
**Ranidae**			
*Rana arvalis* Nilsson, moor frog	5	1.37	3
*Rana ridibunda* Pallas, or *R. esculenta* (L.), marsh or edible frog	20	5.49	12
*Rana* sp., indeterminate frog	45	12.36	13
*Pelophylax lessonae* Camerano, pool frog	10	2.74	5

REPTILIA			
**Emydidae**			
*Emys orbicularis* (L.), European pond terrapin	4	1.09	1
**Lacertidae**			
*Lacerta vivipara* Jacquin, viviparous lizard	2	0.54	1
**Anguidae**			
*Anguis fragilis* L., slow worm	9	2.47	1
**Colubridae**			
*Zamenis longissimus* (Laurenti), Aesculapian snake	3	0.82	1
*Natrix natrix* (L.), grass snake	84	23.07	1
*Natrix tessellata* (Laurenti) or *N. maura* (L.), dice or viperine snake	7	1.92	1
**Viperidae**			
*Vipera berus* (L.), adder	10	2.74	1

AVES			
**Anatidae**			
*Anas platyrhynchos* L., mallard	13	30.95	3
*Anas querquedula* L., garganey duck	1	2.38	1
*Anas strepera* L., gadwall duck	3	7.14	1
*Cygnus cygnus* (L.), whooper swan	2	4.76	1
*Mergus albellus* L., smew	2	4.76	1
**Laridae**			
*Sterna sandvicensis* (Latham), Sandwich tern	1	2.38	1
**Rallidae**			
*Porzana parva* (Scopoli), little crake	2	4.76	1
**Turdidae**			
*Turdus philomelos* Brehm, song thrush	6	14.28	1
*Turdus pilaris* L., fieldfare	6	14.28	1
**Paridae**			
*Parus major* L., 1758, great tit	2	4.76	1
*Parus caeruleus* L. or *P. ater* L., blue or coal tit	2	4.76	1
**Sylviidae**			
*Phylloscopus trochilus* (L.) or *P. collybita* (Viellot), willow warbler or chiffchaff	2	4.76	1

MAMMALIA			
**Insectivora**			
*Sorex* cf. *araneus* L., common shrew	9	0.59	4
*Sorex* cf. *minutus* L., pygmy shrew	4	0.26	2
*Neomys* cf. *browni* Hinton, water shrew	38	2.51	18
*Crocidura* cf. *leucodon* Hermann, bicoloured white-toothed shrew	1	0.06	1
**Chiroptera**			
*Eptesicus serotinus* (Schreber), serotine bat	1	0.06	1
**Primates**			
*Macaca sylvanus* (L.), Barbary macaque	1	0.06	1
**Rodentia**			
*Sciurus vulgaris* L., red squirrel	2	0.13	1
*Castor fiber* L., European beaver	19	1.25	5
*Clethrionomys glareolus* (Schreber), bank vole	191	12.66	46
*Arvicola terrestris cantiana* (Hinton), water vole	563	37.33	60
*Microtus agrestis* (L.), field vole	31	2.05	19
*Microtus agrestis* (L.) or *M. arvalis* (Pallas), field vole or common vole	82	5.43	47
*Microtus* sp., indeterminate vole	503	33.35	106
*Apodemus* cf. *sylvaticus* (L.), wood mouse	42	2.78	9
**Carnivora**			
*Canis lupus* L., wolf	1	0.06	1
*Ursus arctos* L., brown bear	4	0.26	1
*Meles meles* (L.), badger	1	0.06	1
*Mustela* cf. *putorius* L., polecat	3	0.19	2
**Perissodactyla**			
*Equus ferus* Boddaert, horse	1	0.06	1
**Artiodactyla**			
*Capreolus capreolus* (L.), roe deer	11	0.72	2

**Table 6:** Summary of statistical test results (Minitab v.15), using 2-tailed *t*-tests (assuming normal distribution). The results of each duplicate analysis are included in order to provide a statistically significant sample size. Number in “Yes” column represents the number of amino acid (AA) fractions that enable discrimination between the two groups in question at a 95% confidence level. Number in “No” column represents the number of AA fractions that do not enable discrimination between the two sites at a 95% confidence level. For example, in the 2-tailed *t*-tests, analysis of *Bithynia* opercula results in ten out of ten AA fractions support the hypothesis that Cudmore Grove (CG) is distinguishable and younger than the sites correlated with MIS 11. The sites were selected because they have multiple lines of evidence that enable them to be correlated to a specific MIS. Full details of the statistical tests and further stratigraphical information and references associated with the sites are given in Appendix 2. EMR = East Mersea Restaurant Site.

	Sites included in 2-tailed *t*-test	Yes	No
Is CG younger than sites correlated with MIS 11?	Swanscombe, Clacton, Hoxne, Barnham, Elveden, Beeches Pit	10	0
Is CG younger than sites correlated with MIS 9?	Barling, Grays, Hackney, Purfleet	9	1
Is CG older than sites correlated with MIS 7?	Lion Pit, Aveley	8	2
Is EMR younger than sites correlated with MIS 7?	Lion Pit, Aveley	10	0

**Table 7:** Summary of the environmental history of the Pleistocene succession at Cudmore Grove showing details of the fossil groups that have been used in the interpretation of the depositional environments and climate. The deposits with *Hippopotamus* at the ‘East Mersea Restaurant Site’ and the ‘Hippopotamus Site’ (both thought to be MIS 5e in age) are not included.

	
