# Supplementary material for: Differentiation of MIS 9 and MIS 11 in the continental record: vegetational, faunal, aminostratigraphic and sea-level evidence from coastal sites in Essex, UK
Source: Quat Sci Rev. 2009 Nov;28(23-24):2342–73. doi: 10.1016/j.quascirev.2009.04.017 (PMC2806946; doi:10.1016/j.quascirev.2009.04.017)
Supplement: Supplementary file 1 [file mmc1.doc]

Online Supplementary Appendix 1

| NEaar no. | Sample name | Asx D/L | Glx D/L | Ser D/L | Ala D/L | Val D/L | [Ser]/[Ala] |
| --- | --- | --- | --- | --- | --- | --- | --- |
| 0891bF | CGBto1bF | 0.749 ± 0.004 | 0.246 ± 0.002 | 1.010 ± 0.006 | 0.428 ± 0.005 | 0.235 ± 0.002 | 0.413 ± 0.002 |
| 0891bH* | CGBto1bH* | 0.642 ± 0.008 | 0.217 ± 0.001 | 0.703 ± 0.006 | 0.338 ± 0.001 | 0.185 ± 0.000 | 0.403 ± 0.001 |
| 0892bF | CGBto2bF | 0.745 ± 0.002 | 0.260 ± 0.001 | 1.029 ± 0.000 | 0.442 ± 0.004 | 0.227 ± 0.002 | 0.425 ± 0.000 |
| 0892bH* | CGBto2bH* | 0.616 ± 0.003 | 0.204 ± 0.001 | 0.690 ± 0.000 | 0.320 ± 0.001 | 0.173 ± 0.003 | 0.416 ± 0.000 |
| 0893bF | CGBto3bF | 0.751 ± 0.005 | 0.257 ± 0.002 | 1.005 ± 0.021 | 0.446 ± 0.007 | 0.243 ± 0.002 | 0.410 ± 0.002 |
| 0893bH* | CGBto3bH* | 0.648 ± 0.005 | 0.227 ± 0.001 | 0.722 ± 0.001 | 0.363 ± 0.008 | 0.199 ± 0.000 | 0.384 ± 0.004 |
| 0894bF | CGBto4bF | 0.746 ± 0.001 | 0.252 ± 0.003 | 1.011 ± 0.005 | 0.413 ± 0.005 | 0.224 ± 0.002 | 0.408 ± 0.002 |
| 0894bH* | CGBto4bH* | 0.657 ± 0.004 | 0.226 ± 0.000 | 0.750 ± 0.002 | 0.354 ± 0.002 | 0.188 ± 0.001 | 0.388 ± 0.001 |
| 2407bF | EMRBto1bF | 0.610 ± 0.001 | 0.232 ± 0.000 | 0.895 ± 0.006 | 0.240 ± 0.001 | 0.138 ± 0.005 | 0.672 ± 0.002 |
| 2407bH* | EMRBto1bH* | 0.488 ± 0.001 | 0.132 ± 0.003 | 0.432 ± 0.002 | 0.176 ± 0.003 | 0.084 ± 0.005 | 0.617 ± 0.001 |
| 2408bF | EMRBto2bF | 0.596 ± 0.006 | 0.189 ± 0.000 | 0.896 ± 0.004 | 0.210 ± 0.001 | 0.107 ± 0.002 | 0.827 ± 0.002 |
| 2408bH* | EMRBto2bH* | 0.477 ± 0.001 | 0.114 ± 0.000 | 0.495 ± 0.001 | 0.137 ± 0.001 | 0.068 ± 0.000 | 0.629 ± 0.008 |

Amino acid data for *Bithynia tentaculata* opercula from Cudmore Grove (CGB) and the East Mersea Restaurant site (EMRB). Error terms represent one standard deviation about the mean for the duplicate analyses for an individual sample. Each sample was bleached (b), with the free amino acid fraction signified by ‘F’ and the total hydrolysable fraction by ‘H*’.
